# Ximmer: a system for improving accuracy and consistency of CNV calling from exome data

**DOI:** 10.1093/gigascience/giy112

**Published:** 2018-09-06

**Authors:** Simon P Sadedin, Justine A Ellis, Seth L Masters, Alicia Oshlack

**Affiliations:** 1 Bioinformatics, Murdoch Children's Research Institute, Royal Children's Hospital, Flemington Road, Parkville, Victoria 3052 Australia; 2 Victorian Clinical Genetics Services, Royal Children's Hospital, Flemington Road, Parkville, Victoria 3052 Australia; 3 Genes Environment & Complex Disease, Murdoch Children's Research Institute, Royal Children's Hospital Flemington Road, Parkville, Victoria 3052 Australia; 4 Department of Paediatrics, University of Melbourne, Victoria 3010 Australia; 5 Centre for Social and Early Emotional Development, Faculty of Health, Deakin University, Burwood, Victoria 3125 Australia; 6 Inflammation Division, The Walter and Eliza Hall Institute of Medical Research, 1G Royal Parade, Parkville, Victoria 3052, Australia; 7 Department of BioScience, University of Melbourne, Parkville 3050, Australia

**Keywords:** Ximmer: a system for improving exome CNV calling

## Abstract

**Background:**

While exome and targeted next-generation DNA sequencing are primarily used for detecting single nucleotide changes and small indels, detection of copy number variants (CNVs) can provide highly valuable additional information from the data. Although there are dozens of exome CNV detection methods available, these are often difficult to use, and accuracy varies unpredictably between and within datasets.

**Findings:**

We present Ximmer, a tool that supports an end-to-end process for evaluating, tuning, and running analysis methods for detection of CNVs in germline samples. Ximmer includes a simulation framework, implementations of several commonly used CNV detection methods, and a visualization and curation tool that together enable interactive exploration and quality control of CNV results. Using Ximmer, we comprehensively evaluate CNV detection on four datasets using five different detection methods. We show that application of Ximmer can improve accuracy and aid in quality control of CNV detection results. In addition, Ximmer can be used to run analyses and explore CNV results in exome data.

**Conclusions:**

Ximmer offers a comprehensive tool and method for applying and improving accuracy of CNV detection methods for exome data.

## Background

In recent years, high-throughput sequencing (HTS) of DNA has become an essential tool in biomedical science, with a vast range of applications spanning both clinical and research investigations. In clinical settings, whole-exome sequencing (WES) and custom targeted gene panels are especially important and have enabled significant improvements in the rate of diagnosis for rare, genetically heterogeneous disorders [1]. WES has also had a profound impact on disease research by allowing researchers to comprehensively search for protein-altering genetic variation. As a result of these advances, the rate of discovery of new Mendelian disease genes has seen substantial improvements in recent years [2].

While WES has proven highly effective, this success has been based predominantly on the detection of single-nucleotide variants (SNVs) and small insertions and deletions (indels). Larger variants, such as copy number variants (CNVs), are not routinely ascertained from WES data. Nonetheless, CNVs are frequently disease causing, both as the primary genetic lesion for disorders such as α-thalassemia [3], Charcot-Marie-Tooth neuropathy, and Smith-Magenis syndrome, as well as a rare cause for a wide range of Mendelian diseases. In particular, single copy deletions can be pathogenic for any disorder caused by haploinsufficiency. To detect CNVs, patients are often screened for CNVs using SNP or array comparative genomic hybridisation microarrays prior to use of WES. However, affordable microarrays have limited resolution and add time, cost, and complexity to the overall diagnostic workflow. There are consequently significant potential advantages if CNVs can be ascertained directly from WES.

CNVs can be detected from three primary signals in HTS data. These are anomalous mapping of paired-end reads that span CNV breakpoints (PE signals), the splitting of individual reads by CNV breakpoints (split-read, or SR signals), and fluctuation in the coverage of reads falling in the body of a CNV (the read depth, or RD signal). While all of these signals are effective in whole-genome sequencing (WGS) data, the breakpoints of CNVs usually fall between the regions targeted by WES. Therefore, only the RD signal is reliably observable in WES data. The RD signal has been shown to be informative due to a strong correlation of copy number with read coverage depth [4]. However, detection of CNVs is confounded by a range of other factors that also influence read coverage depth. Therefore, these factors must be corrected, and failure to do so can result in significantly degraded accuracy.

Numerous methods have been developed to detect CNVs based on the RD signal. Examples include ExomeDepth [5], ExomeCopy 6, XHMM [7], cn.MOPS [8], ExomeCNV [4], CoNVEX [9], EXCAVATOR [10], CoNIFER [11], CANOES [12], and CODEX [13]. The authors of these tools have often cited high sensitivity and specificity for their methods. However, independent comparisons frequently fail to replicate their findings. For example, Guo et al. reported ExomeDepth having sensitivity of only 19% [14], while Ligt et al. observed a sensitivity of 35% [15]. In the same studies, sensitivity of CoNIFER was cited as being 3% and 29% respectively, compared to the original evaluation estimate of 76%. In some contexts, high accuracy is reported. For example, Jo et al. [16], Ellingford et al. [17], and Feng et al. [18] all cited 100% sensitivity and high specificity for detection of larger CNVs encountered clinically, in each case using high coverage data. However, the circumstances in which high accuracy can be achieved are currently not well understood.

Recent studies have compared performance across multiple datasets [19-21], highlighting the problem of variability in the performance of CNV calling as well as high false-positive rates [22]. Some of the performance variability observed in these studies may be due to differences between the datasets and sequencing design, such as the number of samples, read length, insert size, and mean RD. Also of critical importance is the size and type of CNVs assessed. However, even when these known technical factors are controlled, significant variability is often still observed between datasets.

In this work, we present Ximmer, a software tool that improves CNV calling reliability by enabling users of CNV detection tools to efficiently assess and tune performance. Ximmer contains three parts: a simulation method, an analysis pipeline, and a graphical report. First, Ximmer simulates synthetic single-copy deletions in existing WES data. Then, the analysis pipeline automates detection of the deletions with up to five commonly used CNV detection methods. Finally, the graphical report shows the combined CNV calling results, including a suite of plots that give insight into the accuracy achieved and strategies for improving performance.

Here, we explain the implementation details of Ximmer and demonstrate how using Ximmer improves CNV detection accuracy. We show results from five CNV callers on four datasets representing different exome capture kits and different sequencing depths. Our results concur with previous studies, finding that CNV detection performance is highly variable both within and between datasets. However, we show that using Ximmer to gain insight into the variability enables optimization of the CNV calling and improves detection of real CNVs. Ximmer offers an integrated framework that is easy to use and freely accessible, from [23]. An example of Ximmer output is available at [24].

## Methods

**Figure 1:**
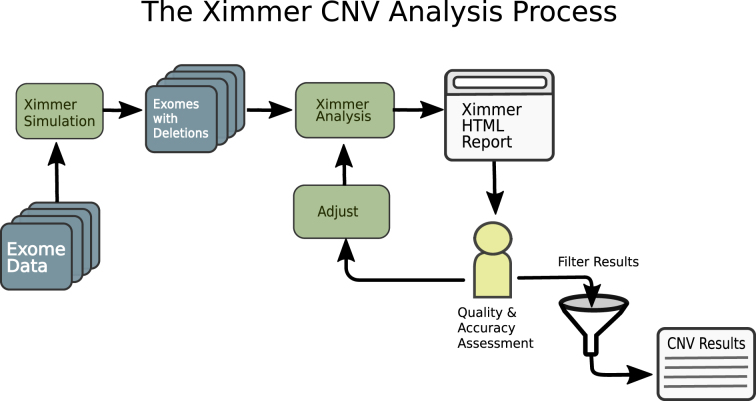
The Ximmer Process. Ximmer consists of three high-level steps. In the first step, simulated CNVs are added to a set of sequence alignments in binary alignment map (BAM) or reference compressed CRAM format. This creates new BAM files containing simulated CNVs that are passed to the integrated analysis pipeline. The analysis pipeline runs up to five different CNV detection methods and collates the results into a graphical report that generates insight into the performance of the tools and possible avenues for improvement. Finally, when the analysis is optimized, it provides an interface to filter CNVs and review and interpret them using the built-in CNV curation tool.

The Ximmer process consists of a series of steps designed to optimize CNV detection performance. The steps consist of: (1) simulation of CNVs in the user's data, (2) execution of CNV callers to find both real and simulated CNVs, (3) quality and accuracy assessment to discover optimal settings for CNV calling, and (4) filtering of results to produce a curated CNV list. This process can be time consuming if conducted manually; however, Ximmer automates all of the steps needed. The high level process is depicted in Fig. 1.

### Simulation

Simulation is the first and most important element of the Ximmer method. By simulating CNVs in the user's own data, Ximmer generates both a prediction of the CNV calling performance and also insights regarding how to improve performance. To simulate CNVs, Ximmer takes advantage of the exclusive use of the RD signal by WES-based CNV detection methods. Specifically, Ximmer removes reads that overlap selected target regions, such that the RD signal is reduced to match the predicted level associated with a single copy deletion. Ximmer focuses on deletions because depleting reads is significantly simpler than realistically synthesizing and adding new reads. For example, synthesized reads would need to accurately reflect the insert size, base quality profile, and alignment characteristics of the original reads. These properties are complex and challenging to model in their own right, requiring intimate knowledge of the specific sample, sequencing technology, and bioinformatic methods applied to the reads. To avoid these difficulties, Ximmer focuses on simulating deletions, which can be simulated purely by removing reads. However, the inferences derived are still likely to apply to other CNV states because, in most CNV calling tools, the same underlying statistical principles are applied regardless of the number of copies being searched for.

The simulation process begins by randomly selecting the genomic region to become the deletion “target.” The reads mapping to these locations can then be depleted using two alternate methods, referred to as “downsampling” and “X-replacement.” The downsampling method randomly removes each read overlapping the deletion target with probability of 0.5, based on an assumption that the relationship between copy number and RD is linear. By contrast, the X-replacement method avoids this assumption. The X-replacement method replaces reads mapping to X chromosome deletion target regions in a female sample with a normalized number of reads from the same genomic regions in a male sample. This method exploits the true difference in copy number between male and female X chromosomes to avoid the assumption of linearity implied by downsampling. The X-replacement method also ensures that other aspects of the reads are preserved in a realistic manner, such as the zygosity and phasing of overlapping SNVs and indels. Further details of the simulation implementation are provided in the Supplementary Methods (S-1). The result of the simulation step is a new set of alignments (binary alignment map [BAM] files) for the whole exome, but with deletions simulated in selected regions.

### CNV analysis pipeline

The second step in the Ximmer process is to analyze the data containing simulated CNVs to produce CNV calls. Ximmer provides a built-in analysis pipeline that automatically installs, configures, and runs five commonly used CNV detection methods. These tools are ExomeDepth, XHMM, cn.MOPS, CoNIFER, and CODEX (see Supplementary Table S1 for an overview of statistical components of these methods). These tools were selected by surveying the literature to ascertain popular methods that are applicable to germline CNV detection. The set was then narrowed to those that were empirically found to be straightforward to install and run reliably within Ximmer's automated framework. We expect to add further tools over time as new methods become available. The analysis pipeline is constructed using Bpipe [25], a framework for creating and running bioinformatic workflows. In addition to running the CNV detection tools, Ximmer performs any necessary pre-processing required by the tools and also post-processing of the results to merge and annotate the resulting CNV calls. Additional CNV callers can be added to Ximmer with only a small effort through the extensible Bpipe framework.

The analysis produces a report in HTML format that contains a full summary of all the simulated deletions, along with a range of plots and tables to highlight CNV calling performance and potential quality issues.

### Results assessment

Once CNV analysis has been performed, the next step is to critically review the HTML report to assess performance of the CNV callers for detecting the simulated deletions and to evaluate options for improving the results.

#### Quality assessment

Three plots are of particular relevance in understanding potential quality issues. These are the sample counts, genome distribution, and quality score calibration plots.

**Figure 2:**
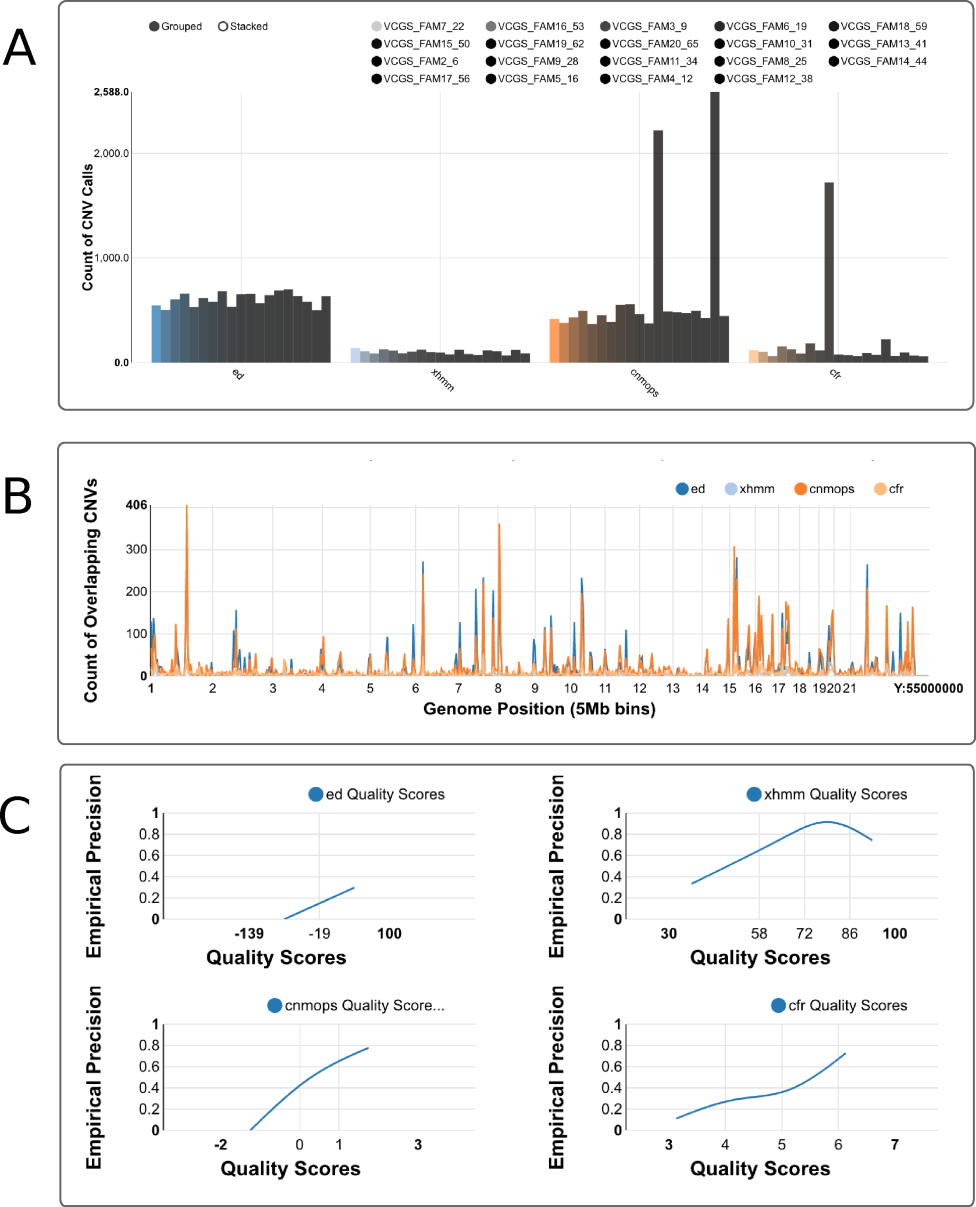
Screen shots of Ximmer quality and accuracy plots. **(A)** Sample counts plot showing the number of CNV calls for each sample, grouped by CNV caller. Calls can be stacked to combine all samples together or split into individual samples using the grouped option. **(B)** Genome distribution plot showing frequency of CNV calls along the genome. **(C)** Quality score calibration plot showing relationship of empirical precision to quality score. These plots may be viewed at full resolution via the example web site [24].

The sample counts plot (Fig. 2A) shows the distribution of the number of CNV calls among the samples, separately for each CNV caller. In most studies, we expect the number of CNV calls to be similar for each sample. If some samples contain a disproportionate fraction of the total CNV calls, it is likely that there is a problem with the sample quality. It may be desirable to remove the samples from the CNV calling altogether, adjust the caller settings to compensate, or isolate poor-quality samples from use in normalizing other samples.

The genome distribution plot (Fig. 2B) divides the genome into 5 Mb bins and displays the number of CNVs overlapping each bin. Clicking on a particular region displays an enlarged plot encompassing that region for more detailed inspection. If particular regions contain very large numbers of CNV calls, it may be desirable to remove these from the target regions used for calling, as their presence may distort quality statistics and degrade overall calling accuracy.

The quality score calibration plot (Fig. 2C) assists in interpreting the confidence measures (or quality scores) assigned to CNVs by the CNV callers. For each caller, Ximmer groups the whole CNV call set into approximately five quality score bins that collectively span the full range of values assigned by the caller. Ximmer then calculates the fraction of calls categorized as true positives in each bin as an estimate of the precision. The estimates are plotted as a line to illustrate the empirical relationship between precision and quality score for each CNV caller. When quality scores are well behaved, it is expected that the precision should increase monotonically as quality score increases. Failure to observe this relationship suggests the caller may produce high-confidence false positives, in which case filtering by quality score alone may be insufficient to reduce the false-positive rate. As with the sample counts plot, it may be appropriate to review normalization settings for methods if quality scores assigned by tools are not well behaved.

#### Accuracy assessment

After reviewing the quality assessment, the next step in the Ximmer process is to review the accuracy estimate. This is presented using a plot designed to mimic a traditional “receiver operator characteristic (ROC)” curve but displayed using absolute measures to better accommodate the unknown positions of true negatives in CNV calling. Instead, the ROC-style plots show how the detection of simulated true positives (*y*-axis) changes with the number of detections not part of the simulation (false positives, *x*-axis) as results are progressively filtered to lower significance levels. It should be noted that false positives are defined as regions that are not simulated to be deletions, but they could actually be true positives from the sample itself. Unlike comparisons of absolute sensitivity and precision, this method primarily compares the ranking of true and false positives and thus takes into account the utility of confidence measures output by tools for filtering the results. For the CNV calling tools included in Ximmer, the confidence measure used for ranking results was chosen in each case by consulting the documentation or by discussion with the tool author (Supplementary Methods, Supplementary Table S2).

The initial display of the ROC-style curve shows the accuracy for the whole set of simulated deletions. As a first step, this can suggest an appropriate level at which to filter results so that the optimal level of sensitivity and specificity is achieved. However, it is frequently of interest to know how sensitivity varies for CNVs of different sizes. The Ximmer accuracy plot can be interactively adjusted to show performance of a subset of CNVs within size ranges specified in base pairs or number of target regions. Further, the accuracy plot can also show the performance of combinations of results such as the intersection or union of results from different CNV callers.

#### CNV discovery

Once the performance of the CNV callers is well understood, the final step in the Ximmer process is to filter the CNV calls according to the decided quality filtering thresholds. This, along with review of the remaining CNVs, can be accomplished using Ximmer's CNV curation interface. The interface combines overlapping CNV calls from different callers into a single merged result. Each merged CNV is listed in an interactive table showing which methods support the CNV call and a range of annotations. The interface supports inspection and filtering by quality scores, overlapping genes, population frequency of relevant CNVs from the database of genomic variants [26], and overlapping SNVs or indels. Additionally, a pictorial diagram is displayed showing the RD deviation over the CNV region and its position relative to overlapping genes.

If desired, the discovery of real CNVs can be determined from the same analysis result set containing simulated CNVs. This approach relies on an assumption that simulated and real CNVs of interest are unlikely to overlap. Alternatively, Ximmer can be re-executed on the original raw data with simulation disabled to derive a stand-alone result set.

### Datasets

To demonstrate the application of Ximmer, we applied it to four datasets representing different Illumina sequencing platforms, exome captures, read configurations, and sequencing depths (Table 1).

**Table 1:** Datasets analyzed with Ximmer

Capture	Samples	Capture size (Mb)	Read length	Mean read depth
SureSelect v5	16 Male, 14 Female	51.2 Mb	2 × 100	30
Nextera 1.2	24 Female, 28 Male	45.3 Mb	2 × 100	120
Nimblegen v2	19 Female, 19 Male	47 Mb	2 × 75	60
TruSeq/Custom Broad Capture	19 Female 16 Male	37.5 Mb	2 × 150	90

The SureSelect dataset was produced as part of an unrelated research program, the Nextera dataset was created as part of the Melbourne Genomics Health Alliance demonstration project [27], and the TruSeq dataset was created by the Broad Institute, Center for Mendelian Genomics. The NimbleGen dataset was downloaded from the Sequence Read Archive (SRA) from a previous study as part of the Simons Foundation Research Autism Initiative [28].

The SureSelect, Nextera, and Nimblegen datasets were analyzed in-house to produce alignment files in BAM format using Cpipe[29]. The TruSeq dataset was produced and analyzed at the Broad Institute using the institute's standard analysis pipeline, also based on GATK.

## Results

### Ximmer simulations

In order to demonstrate Ximmer, we applied it to four different exome datasets with a variety of different properties (Table 2). We configured Ximmer to simulate between 2 and 10 deletions per sample using the X-replacement method in each of the four datasets. As the X-replacement method was employed, deletions were simulated only in the X chromosome of female samples from each respective dataset. The number of simulated CNVs ranged from 72 to 144 for each dataset (see Supplementary Materials). The simulated deletions spanned between 100 bp and 6.9 kbp of targeted bases, equating to genomic spans of between 471 bp and 4.3 Mbp.

**Table 2:** Predicted, actual, and improved sensitivity for validated CNVs from Krumm et al. [30]

Caller	Predicted sensitivity, %	Actual sensitivity, %	Improved sensitivity, %
ExomeDepth	88	90	90 (0)
CODEX	86	96	N/A
XHMM	82	48	76 (+28)
CoNIFER	57	40	48 (+8)
cn.MOPS	24	16	12 (-3)

The predicted sensitivities show the Ximmer estimate of sensitivity based on simulation results. The actual sensitivity shows the empirical sensitivity calculated for validated CNVs in the Nimblegen dataset. Improved sensitivity shows the change in actual sensitivity after adjusting parameters based on Ximmer simulations. The predicted sensitivity is close to the actual sensitivity for all callers except XHMM.

### Comparison of CNV detection methods with default settings

First, we used Ximmer to compare the accuracy of the five different CNV detection methods. Parameters for each tool were set to their defaults, except for cases where the tool setting was clearly misaligned to the simulated data. Specifically, the cn.MOPs minimum CNV width is specified in the manual to be 3 but was lowered to 1 in our analyses. Similarly, the XHMM mean number of targets was lowered to 3. These changes were made to better match the generally smaller size of deletions included in the simulation.

**Figure 3:**
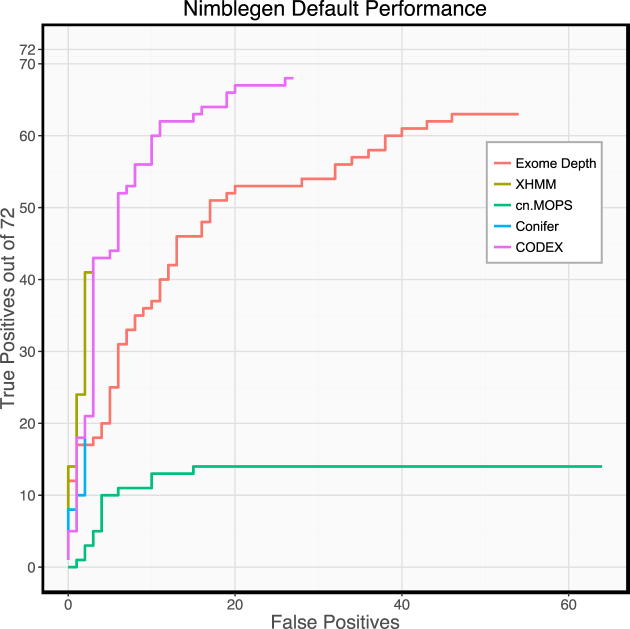
Performance of CNV callers on Nimblegen data with default settings. Performance differs greatly between callers. ExomeDepth and CODEX have significantly higher sensitivity than the other callers, while CoNIFER and XHMM have significantly better precision.

In the Nimblegen dataset, we observe that there were significant differences between the performance of the different CNV callers (Fig. 3). ExomeDepth and CODEX achieved substantially better absolute sensitivity than other tools, finding 88% and 93% of all the simulated deletions, respectively. However, the precision of these tools was poor (54% and 72%) compared to XHMM (93%). For ExomeDepth, a substantial difference in precision persisted even when results were filtered to yield the same sensitivity as XHMM. Therefore, in this case, CODEX appears to be the optimal CNV caller. Both cn.MOPs and CoNIFER performed poorly in terms of sensitivity, each finding less than 30% of simulated deletions. cn.MOPs has very poor precision in this dataset (0.5%) and appears to output many very high confidence calls that are ranked higher than the true positives it detects.

### Comparison between datasets

**Figure 4:**
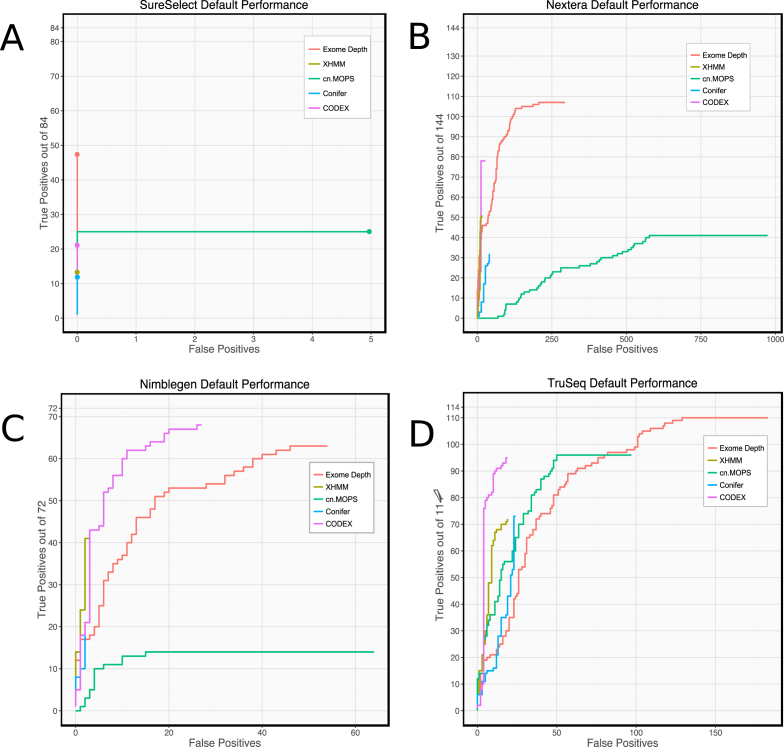
ROC-style curves with default parameters. Count of true positives vs false positives as ranked results are filtered by varying the quality score threshold, when the five CNV calling methods are executed on four different datasets with their default parameters. Performance is highly variable both between different methods on the same dataset and between the same method on different datasets.

Next, we compared Ximmer results using the five CNV callers with default settings on all four datasets. Our results (Fig. 4) show that individual methods have marked differences in performance on different datasets. For example, all callers exhibited a low false-positive rate when applied to the SureSelect data (fewer than 10 false-positive calls for any caller) but showed much higher false-positive rates on Nextera data (ExomeDepth and cn.MOPs both having more than 200 false-positive calls). cn.MOPs performed poorly on the Nimblegen and Nextera data, detecting very few true and many false CNVs. However, cn.MOPs performed well with TruSeq data, having better sensitivity than XHMM and CoNIFER and better precision than ExomeDepth. These differences suggest that some datasets are better suited to the algorithms or default settings of particular calling methods.

Despite the differences, some aspects of individual caller performance were mostly consistent across datasets. XHMM and CODEX were consistently more precise than other callers, and ExomeDepth achieved higher total sensitivity than any other caller in all datasets except the Nimblegen data.

In some respects, differences between datasets are consistent between callers. With SureSelect data (30x mean coverage), no caller could achieve more than 60% sensitivity. However, with TruSeq data (90x mean coverage), all callers found more than 60% of the simulated deletions, and ExomeDepth found nearly all deletions (96%).

It is likely that homogeneity of the data is an important factor in determining these characteristics. Data sets having very low intersample variation with few batch effects may work well with callers that apply relatively little normalization or are flexible in their normalization approach.

Overall, our results suggest that each dataset has individual characteristics that affect the performance of each CNV caller differently. Consequently, there is no single best CNV detection tool for all datasets. Depending on the priorities of the investigation and the particular dataset in question, a different tool or combination of tools may be more appropriate. Therefore, users should assess their own data and choose CNV detection methods using Ximmer.

### CNV calling performance can be improved with parameter optimization

Next, we configured Ximmer to re-analyze the Nimblegen data using slightly larger simulated deletions (4–15 target regions), while varying several configurable parameters for ExomeDepth, XHMM, cn.MOPs, and CoNIFER. The parameters that were varied were chosen by reviewing the documentation and experimenting to find those with the largest direct effect on sensitivity (Supplementary Table S3). We did not include CODEX in this analysis because it automatically tunes its main parameter (K, number of latent factors) using an iterative procedure.

**Figure 5:**
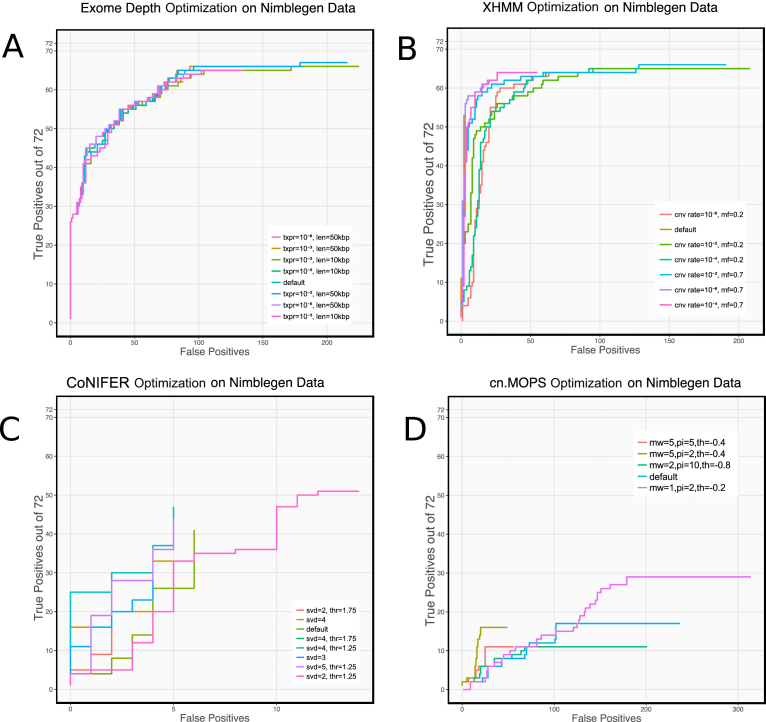
Results of adjusting CNV calling parameters on ROC-style curves. XHMM, CoNIFER, and cn.MOPs all have configurations where sensitivity or precision can be substantially improved. Reducing CoNIFER calling threshold to 1.25 increases sensitivity from 25% to 40%. Increasing the exome-wide CNV rate to 10^−4^ and reducing the normalization factor from 0.7 to 0.2 increases XHMM sensitivity from 67% to 88% Reducing the cn.MOPs prior impact factor to 2 and raising the calling threshold to -0.4 allowed sensitivity to nearly double (from 24% to 36%); however, these settings caused a substantial reduction in precision.

We found that adjusting two parameters (the exome-wide CNV rate to 10^−4^ and the normalization factor to 0.2) increased XHMM sensitivity (Fig. 5B) by 21% (67% to 88%) with an acceptable loss of precision (81% to 55%). Similarly, we evaluated alternative values for the singular value decomposition (SVD) number and calling threshold for CoNIFER (Fig. 5C) and found that by adjusting the calling threshold parameter from 1.5 to 1.25, sensitivity could be improved by 25% to 40% with only a small sacrifice in precision. cn.MOPs adjustments were able to improve sensitivity from 19% to 36% by adjusting the prior impact parameter from 5 to 2 and by adjusting the calling threshold upward from -0.8 to -0.4. Although we tried varying two parameters (transition probability and expected CNV length), ExomeDepth appeared to have nearly optimal parameters as its defaults for this dataset.

This analysis demonstrates that tuning parameter settings should be considered an important element of using CNV detection tools and can lead to significantly improved accuracy. Many previous comparison studies [19,20] have evaluated CNV methods without rigorous optimization of parameters. Our results suggest that the discrepancies in the results from these studies may have been reduced if calling parameters were optimized.

### Optimization of parameters across datasets

**Figure 6:**
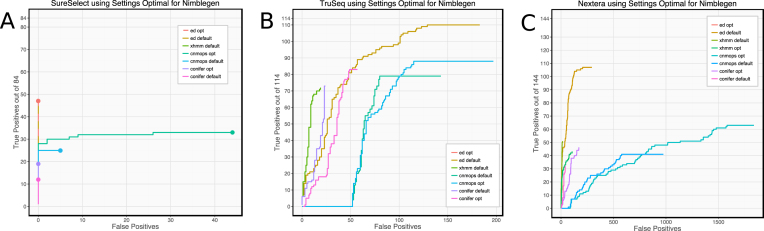
Performance of other datasets (A: SureSelect, B: TruSeq, C: Nextera) when analyzed using parameters optimal for Nimblegen data (opt) compared to default settings (default). Nimblegen-optimized parameters are frequently unsuitable on other datasets. XHMM is severely compromised by the Nimblegen settings on all datasets. SureSelect and TruSeq data produce no XHMM CNV calls, while both sensitivity and precision are poorer in Nextera data. CoNIFER and cn.MOPs both gain in sensitivity but by a much smaller proportion and with a larger inflation of false-positive calls than with Nimblegen data.

We applied the optimized settings derived from simulation performance on Nimblegen data to the analysis of the other three datasets. However, we observed that these settings are not optimal for every other dataset. For example, on the SureSelect and TruSeq data (Fig. 6A and B), XHMM achieves sensitivity of 63% and precision of 80% with the default settings but produces no calls at all with the optimized settings from the Nimblegen dataset. The optimizations increase sensitivity in cn.MOPs; however, the marginal increase (69% to 77%) is much less significant than for Nimblegen data and causes a substantially higher number of false-positive calls. Similarly, CoNIFER also shows a much smaller proportional increase in sensitivity (64% to 73%) and experiences a significant fall in precision (75% to 59%).

We conclude that optimization needs to be performed on each dataset or data type separately. Ximmer supports this process efficiently and easily through modification of simple configuration settings.

### Application of Ximmer to a set of validated CNVs

We extracted a set of validated CNVs for the samples that were captured in the Nimblegen dataset from a previous study by N. Krumm et al. [30]. After filtering to include only CNVs overlapping autosomal target regions of the exome capture, 25 validated CNVs remained. We then tested detection of these CNVs from the exome data using Ximmer, first using default parameters as described above for each of the five CNV callers. With the exception of XHMM, the sensitivity estimated by Ximmer using simulation approximately reflected the sensitivity observed on the validated CNVs (Table 2). In the case of XHMM, we suspect that differences in the composition of the CNV sizes between the simulation and the validated CNV set may partially account for the discrepancy. Precision is harder to evaluate as predictions of CNVs in regions not in our validated set could be true deletions, and the false-negative rate in the validated results is uncertain. However, the number of total detections varied greatly between callers (CoNIFER and XHMM had fewer than 12 compared to ExomeDepth and cn.MOPS, which had more than 200), as predicted by Ximmer.

Next, we applied the optimized settings identified previously through simulation to improve sensitivity for ExomeDepth, XHMM, and ConNIFER. Due to the poor precision observed with the default settings for cn.MOPS, we chose to improve precision rather than sensitivity. By reviewing the sample counts plot, we determined that a significant fraction of the putative false-positive calls were concentrated in just 3 of 20 samples (Supplementary Fig. S7). Therefore, we excluded these samples from the analysis for cn.MOPS.

Incorporation of parameter adjustments suggested by Ximmer resulted in substantially improved performance of several methods. For example, sensitivity was improved in XHMM (+28%) and CoNIFER (+8%). Conversely, removing the three poor-quality samples from cn.MOPs slightly lowered sensitivity but removed 90% (856) of the false-positive CNV calls.

## Conclusion

While there is great utility in detecting CNVs from WES data, adoption of CNV detection methods in practice has met with significant challenges. These are primarily centered around highly unpredictable performance and lack of reproducibility between datasets. We have addressed these challenges by creating Ximmer, a tool that facilitates efficiently assessing and improving the accuracy of WES-based CNV detection methods. Our comparison of four different datasets analyzed by five different CNV calling methods represents one of the most comprehensive evaluations to date. Our results show, consistent with previous studies, that there is significant variability in performance of CNV detection between tools and between datasets. We conclude that to effectively use these methods, attention must be given to understand and optimize their behavior on each individual dataset. Ximmer can be used to automate these procedures, avoiding a significant burden. In addition, we have demonstrated that Ximmer can produce valuable insights into the quality of datasets for CNV calling and the behavior of CNV detection tools. While Ximmer's simulation framework focuses on heterozygous deletions, the evaluation framework supports all copy number states, which could be simulated using different simulation methods, supplied using real true-positive samples, or implemented as a future extension. We note, however, that homozygous and hemizygous CNVs are generally significantly easier to characterize than heterozygous states and thus are of less interest as subjects of simulation. Another valuable extension would be simulation and evaluation of CNV calling in WGS datasets. WGS, however, requires different simulation methods because WGS methods typically harness breakpoint signals that are not used in exome analysis. As the first tool offering combined simulation, evaluation, tuning, and interpretation of results from CNV detection methods, we believe Ximmer will assist with increasing practical adoption of CNV detection methods for exome data. Ximmer is open source and available at [23]. An example Ximmer report can be viewed online at [24].

## Availability of source code and requirements

Project name: Ximmer

Project home page: http://ximmer.org

Operating system: Linux/Unix

Programming language: Groovy, R, Python

Other requirements: Java 1.8

License: LGPL

Research Resource Identifier (RRID): RRID:SCR_016427

## Availability of supporting data

The SureSelect, Nimblegen, and Nextera datasets are available from SRA under accession numbers SRP132744, SRP010920, and SRP148622, respectively. The NimbleGen dataset is available from dbGaP under accession number phs001272. Data further supporting this work are also openly available in the GigaScience repository, GigaDB [31].

## Additional files

**Additional File 1**. A set of figures, tables and supplementary methods supporting the results. Details of the simulation method. Description of methodology for optimising tuning parameters of data sets.

**Supplementary Figure S1**. Ximmer downsampling method. A set of adjacent target regions is chosen, and reads aligning to the chosen target regions are randomly removed with probability 0.5. Although the read coverage is depleted similarly to a deletion, the reads come from both alleles rather than a single allele.

**Supplementary Figure S2**. Ximmer X-replacement Method—A set of adjacent X chromosome target regions are selected and expanded to define a region having no overlapping reads at each end as the deletion region. All reads overlapping the deletion region are removed from a female sample, and a library-size normalised number of reads from a male sample are added in their place. By replacing female reads with those from a male, Ximmer utilises the difference in ploidy of the X-chromosome to simulation single copy deletion without arbitrarily downsampling reads. While downsampling is still applied in the X-replacement method, it is only used to compensate for library size differences between the two samples.

**Supplementary Figure S3**. Optimisation of calling parameters for ExomeDepth. Transition probability (txpr) and expected CNV length (len) were adjusted. Although significant variations were applied to these parameters, ExomeDepth performance did not change significantly.

**Supplementary Figure S4**. Optimisation of calling parameters for XHMM. Exome-wide CNV rate (cnv rate) and normalisation mean factor (mf) were adjusted. By adjusting the cnv rate to 10^–4^ and the normalisation mean factor to 0.2, sensitivity could be improved without significant loss of precision.

**Supplementary Figure S5**. Optimisation of calling parameters for Conifer. SVD number and calling threshold were adjusted to non-default values (see legend). Changing SVD number to 4 and using threshold 1.25 slightly increased sensitivity, however greater increase was observed with the default SVD number (2) and calling threshold of 1.25.

**Supplementary Figure S6**. Optimisation of calling parameters for cn.MOPs. Three parameters were adjusted through a range of values to find the optimal settings: minimum width (mw) from 1 to 4, prior impact (pi) from 2 to 10, and calling threshold (th) from -0.2 to -0.8. The settings of minimum width=2, prior impact=2 and calling threshold=-0.2 offer nearly double the sensitivity (increase from 24% to 40%), however these settings cause a substantial degradation in precision.

**Supplementary Figure S7**. Sample count QC plot showing disproportionate numbers of CNV calls concentrated in 3 particular samples across all parameter settings. Each separate configuration of cn.MOPs is represented by a separate group of bars along the x-axis. Within each group, three bars can be seen to significantly exceed the height of other bars, representing poor quality samples within the batch.

**Supplementary Table S1**. Comparison of implementation details for a selection of read-depth (RD) based CNV detection methods.CNV detection methods. Most RD methods consist of three stages: normalisation to remove unwanted variation, statistical modeling of the residual variation and segmentation of the genome into regions of contiguous copy number.

**Supplementary Table S2**. Confidence measures chosen for each tool for ranking and filtering CNVs in Ximmer's accuracy assessment

**Supplementary Table S3**. For each CNV caller various parameters were chosen to vary for optimisation across a range of values on the Nimblegen data set.

## Abbreviations

BAM: Binary alignment map; CGH: Comparative genomic hybridisation; CNV: copy number variant; CRAM: Reference compressed alignment file; HTS: high-throughput sequencing; indels: insertions and deletions; PE: paired-end; QC: quality control; RD: read depth; ROC: receiver operator characteristic; SNV: single-nucleotide variant; SR: split-read; WES: whole-exome sequencing; WGS: whole-genome sequencing.

## Ethics, consent, and permissions

Generation of sequencing data for the SureSelect data was approved by the Royal Children's Hospital (Melbourne) Human Research Ethics Committee under approval HREC #27 127. Other datasets were derived from publicly available data governed by ethics arrangements associated with the originating studies.Informed written consent was obtained from all individuals.

## Competing interests

The authors declare that they have no competing interests.

## Funding

Sequencing for the TruSeq dataset was provided by the Center for Mendelian Genomics at the Broad Institute of the Massachusetts Institute of Technology and Harvard University and was funded by the National Human Genome Research Institute, the National Eye Institute, and the National Heart, Lung, and Blood Institute (grant UM1 HG008900) to Daniel MacArthur and Heidi Rehm.

## Author contributions

S.S. conceived of and implemented the method, performed simulation work, and drafted the manuscript. A.O. advised on design, implementation, and interpretation of results and edited the manuscript. S.M. and J.E. collected samples, extracted DNA, arranged sequencing, and provided feedback on the manuscript. All authors approved the final manuscript.

## Supplementary Material

GIGA-D-18-00165_Original_Submission.pdf

GIGA-D-18-00165_Revision_1.pdf

Response_to_Reviewer_Comments_Original_Submission.pdf

Reviewer_1_Report_(Original_Submission) -- Yuchao Jiang 6/6/2018 Reviewed

Reviewer_1_Report_Revision_1 -- Yuchao Jiang 8/8/2018 Reviewed

Reviewer_2_Report_(Original_Submission) -- Fatemeh Zare 7/6/2018 Reviewed

Reviewer_3_Report_(Original_Submission) -- Yuval Itan 6/23/2018 Reviewed

Reviewer_3_Report_Revision_1 -- Yuval Itan 8/10/2018 Reviewed

Reviewer_4_Report_(Original_Submission) -- Je-Gun Joung 6/24/2018 Reviewed

Reviewer_4_Report_Revision_1 -- Je-Gun Joung 8/13/2018 Reviewed

Supplemental Files

## References

[1] Stark Z, Tan TY, Chong B A prospective evaluation of whole-exome sequencing as a first-tier molecular test in infants with suspected monogenic disorders. Genet Med. 2016;18(11):1090–6. 26938784 10.1038/gim.2016.1

[2] Zhang X Exome sequencing greatly expedites the progressive research of Mendelian diseases. Front Med. 2014;8(1):42–57. 24384736 10.1007/s11684-014-0303-9

[3] Stankiewicz P.and Lupski JR.(2010) Structural variation in the human genome and its role in disease. Annu. Rev. Med. 61, 437–55. 10.1146/annurev-med-100708-20473520059347 20059347

[4] Sathirapongsasuti JF, Lee H, Horst BA, Exome sequencing-based copy-number variation and loss of heterozygosity detection: ExomeCNV. Bioinformatics. 2011;27(19):2648–54. 21828086 10.1093/bioinformatics/btr462 PMC3179661

[5] Plagnol V, Curtis J, Epstein M A robust model for read count data in exome sequencing experiments and implications for copy number variant calling. Bioinformatics. 2012;28:2747–54. 22942019 10.1093/bioinformatics/bts526 PMC3476336

[6] Love MI., Myšičková A., Sun R., Kalscheuer V., Vingron M.and Haas SA.(2011) Modeling read counts for CNV detection in exome sequencing data. Stat Appl Genet Mol Biol 10, 10.2202/1544-6115.173223089826 PMC3517018 23089826

[7] Fromer M, Moran JL, Chambert K Discovery and statistical genotyping of copy-number variation from whole-exome sequencing depth. Am J Hum Genet. 2012;91:597–607. 23040492 10.1016/j.ajhg.2012.08.005 PMC3484655

[8] Klambauer G, Schwarzbauer K, Mayr A, cn.MOPS: mixture of Poissons for discovering copy number variations in next-generation sequencing data with a low false discovery rate. Nucleic Acids Res. 2012;40:e69. 22302147 10.1093/nar/gks003 PMC3351174

[9] Amarasinghe KC., Li J.and Halgamuge SK.(2013) CoNVEX: copy number variation estimation in exome sequencing data using HMM. BMC Bioinformatics 14 Suppl 2, S2 10.1186/1471-2105-14-S2-S223368785 PMC3549847 23368785

[10] Magi A, Tattini L, Cifola I, EXCAVATOR: detecting copy number variants from whole-exome sequencing data. Genome Biol. 2013;14:R120. 24172663 10.1186/gb-2013-14-10-r120 PMC4053953

[11] Krumm N, Sudmant PH, Ko A Copy number variation detection and genotyping from exome sequence data. Genome Res. 2012;22:1525–32. 22585873 10.1101/gr.138115.112 PMC3409265

[12] Backenroth D, Homsy J, Murillo LR, CANOES: detecting rare copy number variants from whole exome sequencing data. Nucleic Acids Res. 2014;42:e97. 24771342 10.1093/nar/gku345 PMC4081054

[13] Jiang Y, Oldridge DA, Diskin SJ, CODEX: a normalization and copy number variation detection method for whole exome sequencing. Nucleic Acids Res. 2015;43:e39. 25618849 10.1093/nar/gku1363 PMC4381046

[14] Guo Y, Sheng Q, Samuels D Comparative study of exome copy number variation estimation tools using array comparative genomic hybridization as control. BioMed Res. 2013;2013:7. 10.1155/2013/915636 PMC3835197 24303503

[15] de Ligt J, Boone PM, Pfundt R Detection of clinically relevant copy number variants with whole-exome sequencing. Hum Mutat. 2013;34:1439–48. 23893877 10.1002/humu.22387

[16] Jo H-Y, Park M-H, Woo H-M, Application of whole-exome sequencing for detecting copy number variants in CMT1A/HNPP. Clin Genet. 2016;90:177–81. 26662885 10.1111/cge.12714

[17] Ellingford JM, Campbell C, Barton S Validation of copy number variation analysis for next-generation sequencing diagnostics. Eur J Hum Genet. 2017;25:719–24. 28378820 10.1038/ejhg.2017.42 PMC5427176

[18] Feng Y, Chen D, Wang G-L Improved molecular diagnosis by the detection of exonic deletions with target gene capture and deep sequencing. Genet Med. 2014;17:1–9. 10.1038/gim.2014.80 PMC4338802 25032985

[19] Hong CS, Singh LN, Mullikin JC, Assessing the reproducibility of exome copy number variations predictions. Genome Med. 2016;8:82. 27503473 10.1186/s13073-016-0336-6 PMC4976506

[20] Tan R, Wang Y, Kleinstein SE An evaluation of copy number variation detection tools from whole-exome sequencing data. Hum Mutat. 2014;35:899–907. 24599517 10.1002/humu.22537

[21] Zare F, Dow M, Monteleone N An evaluation of copy number variation detection tools for cancer using whole exome sequencing data. BMC Bioinforma. 2017;18:286. 10.1186/s12859-017-1705-x PMC5452530 28569140

[22] Samarakoon PS, Sorte HS, Stray-Pedersen A, cnvScan: a CNV screening and annotation tool to improve the clinical utility of computational CNV prediction from exome sequencing data. BMC Genomics. 2016;17:51. 26764020 10.1186/s12864-016-2374-2 PMC4712464

[23] http://ximmer.org, "Ximmer User Guide"

[24] http://example.ximmer.org, "Example Ximmer Report"

[25] Sadedin SP, Pope B, Oshlack A Bpipe: a tool for running and managing bioinformatics pipelines. Bioinformatics. 2012;28:1525–6. 22500002 10.1093/bioinformatics/bts167

[26] Zhang J, Feuk L, Duggan GE Development of bioinformatics resources for display and analysis of copy number and other structural variants in the human genome. Cytogenet Genome Res. 2006;115:205–14. 17124402 10.1159/000095916

[27] https://www.melbournegenomics.org.au/, "Melbourne Genomics Health Alliance"

[28] Sanders SJ, Murtha MT, Gupta AR De novo mutations revealed by whole-exome sequencing are strongly associated with autism. Nature. 2012;485:237–41. 22495306 10.1038/nature10945 PMC3667984

[29] Sadedin SP., Dashnow H., James PA., Bahlo M., Bauer DC., Lonie A., Lunke S., Macciocca I., Ross JP., Siemering KR., Stark Z., White SM., Taylor G., Gaff C., Oshlack A.and Thorne NP.(2015) Cpipe: a shared variant detection pipeline designed for diagnostic settings. Genome Med 7, 68 10.1186/s13073-015-0191-x26217397 PMC4515933 26217397

[30] Krumm N., Turner TN., Baker C., Vives L., Mohajeri K., Witherspoon K., Raja A., Coe BP., Stessman HA., He ZX., Leal SM., Bernier R.and Eichler EE.(2015) Excess of rare, inherited truncating mutations in autism. Nat. Genet. 47, 582–8. 10.1038/ng.330325961944 PMC4449286 25961944

[31] Sadedin SP, Ellis JA, Masters SL Supporting data for “Ximmer: a system for improving accuracy and consistency of CNV calling from exome data.”. GigaScience Database. 2018 10.5524/100495. PMC6177737 30192941

