# Supplementary material for: Ximmer: a system for improving accuracy and consistency of CNV calling from exome data
Source: Gigascience. 2018 Sep 6;7(10):giy112. doi: 10.1093/gigascience/giy112 (PMC6177737; doi:10.1093/gigascience/giy112)
Supplement: Supplemental Files [file giy112_supplemental_files.zip › Ximmer_Supplementary_Material2_Final.docx]

# Ximmer: a system for improved results from CNV Calling from Exome Data: Supplementary Material

## 1 : Additional Details of Simulation Methods

Ximmer supports two different methods for simulating CNVs. These are referred to respectively as “downsampling” and “X-replacement”. In both cases, Ximmer exploits the exclusive use of the RD signal by WES based CNV detection methods. Due to their reliance on the RD signal, CNVs can be simulated by adding or removing reads to an existing alignment to influence the signal in a similar manner as a CNV.

While this principle applies to all CNV types, simulation of amplifications is significantly more difficult than deletions, because it requires new reads to be added. To be realistic, the added reads should include overlapping variants, read errors, mapping errors, contamination and other distortions. Inclusion of all these factors into Ximmer would substantially complicate the method. However in contrast to amplifications, deletions may be simulated purely by removing reads from the alignment, which avoids all the complexity of read synthesis. To simplify read simulation, Ximmer simulates only deletions for evaluation and tuning of CNV detection methods.

**Simulation by Downsampling**

The goal of both simulation methods is to create a new alignment where the deletion region is depleted of reads to mirror the effect of a real CNV. The downsampling method achieves this by directly removing a fixed proportion of reads from an alignment over a selection of adjacent target regions. This is the first simulation method offered by Ximmer and has been frequently employed by tool authors in evaluation of their methods. For example downsampling was applied to simulate CNVs in the case of CoNIFER, CONTRA, and CoNVEX. In practice, the proportion of reads removed is often simply a half, based on the assumption that the relationship between read counts and true DNA copy number is linear. While simple, downsampling in this manner takes no account of any potential non-linearities that may skew read coverage away from half the original value. Since the assumption of linearity is often heavily relied on by CNV callers, overly optimistic results could be produced when simulating using this method. A separate problem is that down-sampling is usually implemented without phasing to a particular haplotype. As a result, heterozygous variants may be observed in the simulated deletions, where these should exhibit a complete loss of heterozygosity.


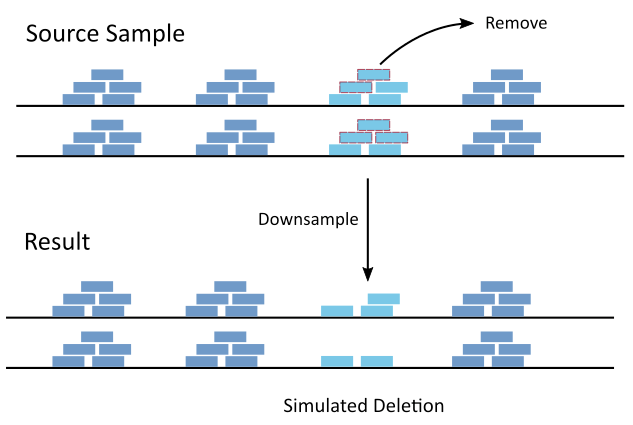


***Figure S1****: Ximmer downsampling method. A set of adjacent target regions is chosen, and reads aligning to the chosen target regions are randomly removed with probability 0.5. Although the read coverage is depleted similarly to a deletion, the reads come from both alleles rather than a single allele.*

**Simulation by X-Replacement**

Due to the limitations of downsampling, we sought to offer an alternative simulation method in Ximmer. The method, referred to as “X-Replacement”, avoids the assumption of linearity by exploiting the true difference in copy number between males and females on the X chromosome. A deletion is simulated by completely removing all reads mapping to a set of adjacent X chromosome target regions in a female sample. Reads mapping to the same target regions in a male sample are then inserted in their place (Figure 3). The number of reads inserted is adjusted only for library size and thus carries a true signal representing the difference between read counts for haploid and diploid DNA in the chosen regions. X-replacement incurs some drawbacks compared to downsampling including not accounting for sample specific effects such as GC bias. However we believe that it offers a valuable alternative because it preserves many of the biases in read counts that may lead to poorer performance of CNV detection methods. Ximmer offers both methods as options.


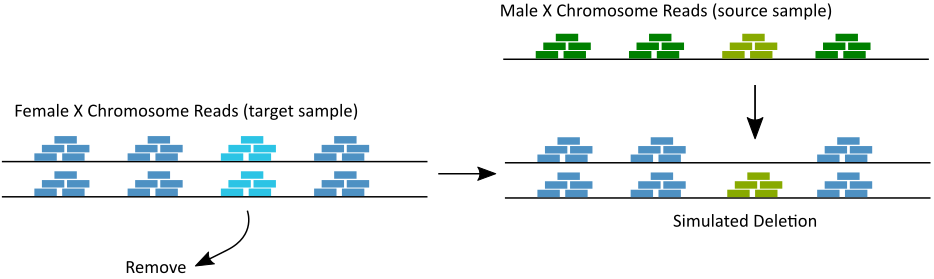


***Figure S2:*** *Ximmer X-replacement Method - A set of adjacent X chromosome target regions are selected and expanded to define a region having no overlapping reads at each end as the deletion region. All reads overlapping the deletion region are removed from a female sample, and a library-size normalised number of reads from a male sample are added in their place. By replacing female reads with those from a male, Ximmer utilises the difference in ploidy of the X-chromosome to simulation single copy deletion without arbitrarily downsampling reads.* *While downsampling is still applied in the X-replacement method, it is only used to compensate for library size differences between the two samples.*

**Additional Considerations**

Both the downsampling and X-replacement methods require several additional considerations. The first is that read coverage does not always fall to zero at the point where Ximmer initially chooses to begin or end a simulated deletion. This would require Ximmer to simulate complex edge effects of the deletion in the reads split by the deletion boundary. To avoid this complexity, Ximmer simply expands the edges of a simulated deletion until a gap in read coverage is encountered. This process slightly inflates the size of deletions.

Another consideration is the possibility that simulated deletions will overlap real CNVs present in the sample. To prevent this, Ximmer firstly ensures that no two simulated CNVs overlap. Secondly, Ximmer prevents overlap with population CNVs by avoiding simulation in any region overlapping a CNV with a frequency greater than 1% in the Database of Genomic Variants (DGV).

## 2: CNV Calling Evaluation

### Versions of CNV Callers

| **Tool / Method** | **Version** |
| --- | --- |
| XHMM | 1.0 |
| ExomeDepth | 1.1.10 |
| cn.MOPS | 1.16.2 (*) |
| ConiFER | 0.2.2 |
| CODEX | 1.0 |

(*) This version is no longer compatible with current versions of Bioconductor. To reproduce results, use Bioconductor 3.2 or install version 1.16.2 from the following github repository:

<https://github.com/ssadedin/cn.mops/tree/1.16.2-port-forward>

This version has minimal changes to allow operation with modern version of Bioconductor.

### CNV Caller Properties

Supplementary Table S1 *Comparison of implementation details for a selection of read-depth (RD) based CNV detection methods.CNV detection methods. Most RD methods consist of three stages: normalisation to remove unwanted variation, statistical modeling of the residual variation and segmentation of the genome into regions of contiguous copy number.*

| **Tool / Method** | **Normalisation** | **Statistical Model** | **Segmentation** |
| --- | --- | --- | --- |
| XHMM | PCA / SVD | Normal Distribution | Hidden Markov Model |
| ExomeDepth | PCA/ SVD | Negative Binomial | Hidden Markov Model |
| cn.MOPS | Median (simple) | Poisson | Fastseg; similar to Circular binary segmentation |
| ConiFER | PCA / SVD | Normal Distribution (z-score) | Simple joining of adjacent regions |
| CODEX | Iterative PCA | Log-linear Poisson | Circular Binary Segmentation |

### Confidence Measures

*Table S2: Confidence measures chosen for each tool for ranking and filtering CNVs in Ximmer’s accuracy assessement*

| **Caller** | **Confidence Measure** | **Description** |
| --- | --- | --- |
| XHMM | Q_SOME | Phred scaled likelihood that a CNV is present overlapping the call |
| ExomeDepth | Beta factor | Phred scaled likelihood that deletion is real |
| Cn.MOPs | I/NI | Ratio of informative and non-informative regions within CNV call |
| Conifer | log_10_(CNV Size in bp) | Conifer does not provide a direct confidence measure for each CNV. As a proxy for a confidence measure, the log-scaled size (in base pairs) of CNV is used. |

### True / False Positive Categorisation

Ximmer categorizes calls as “true positives” if they overlap with a simulated deletion or with a known true positive provided in the configuration. Calls are classified “false positives” if there is no overlap. In some cases, events categorized as “false positives” may be true CNVs in the original samples. To reduce the frequency of this occurrence, calls from the false positive set that overlap a variant found in the Database of Genomic Variants (DGV) (J. Zhang et al. 2006) at a frequency greater than 1% are discarded.

## Optimisation of CNV Calling Methods

### Parameters Selected for Optimisation

Table S3 *For each CNV caller various parameters were chosen to vary for optimisation across a range of values on the Nimblegen data set*.

| Caller | Parameter | Range / Values (Default) |
| --- | --- | --- |
| XHMM | Exome Wide CNV Rate | 10^-2^ - 10^-8^ (10^-8^) |
|  | Normalisation Factor | 0.2, 0.7 (0.7) |
| Exome Depth | Transition Probability | 10^-1^ - 10^-6^  (10^-4^) |
|  | Expected CNV Length | 10kbp, 50kbp |
| cn.MOPS | Prior Impact | 1 - 10 (5) |
|  | Minimum Width | 1,2,4 (1) |
|  | Calling Threshold | -0.2, -0.4, -0.8 (-0.8) |
| Conifer | SVD Number | 1 - 8 (2) |
|  | Calling Threshold | 1.25, 1.5, 1.75 (5) |

### Optimisation of ExomeDepth Calling Parameters

ExomeDepth parameters were optimised by adjusting two parameters: the “transition probability”, representing a prior probability of transitioning into a non-diploid state; and the expected CNV length.


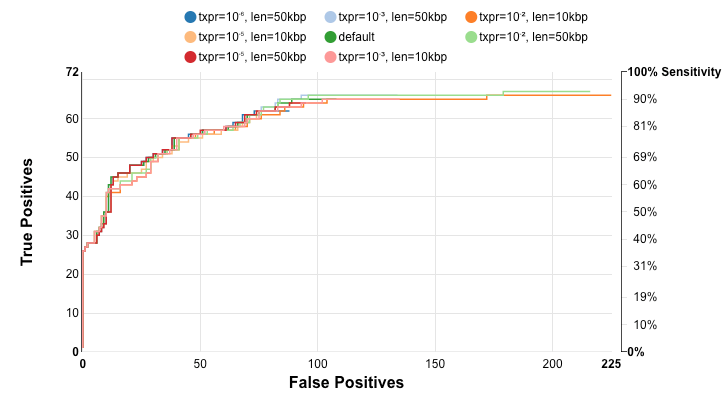


***Figure S3:*** *Optimisation of calling parameters for ExomeDepth. Transition probability (txpr) and expected CNV length (len) were adjusted. Although significant variations were applied to these parameters, ExomeDepth performance did not change significantly.*

### Optimisation of XHMM Calling Parameters

Two parameters were explored for adjusting XHMM performance: the exome wide cnv rate (or “cnv rate”), and the “mean factor” representing the fraction of variation used to choose the number of singular value decomposition components to remove from the data.


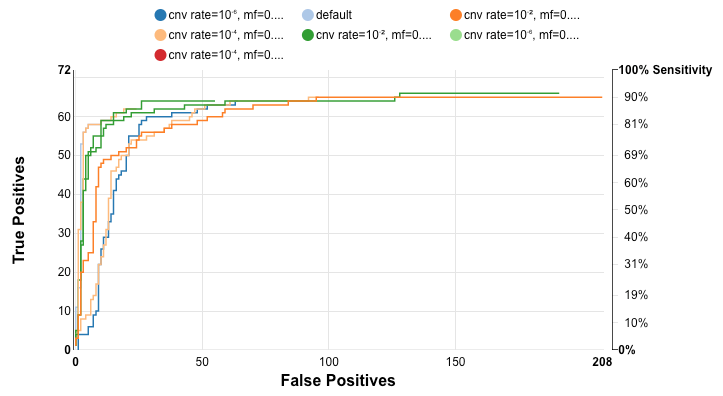


***Figure S4:*** *Optimisation of calling parameters for XHMM. Exome-wide CNV rate (cnv rate) and normalisation mean factor (mf) were adjusted. By adjusting the cnv rate to 10^-4^ and the normalisation mean factor to 0.2, sensitivity could be improved without significant loss of precision.*

### Optimisation of Conifer Calling Parameters

Two calling parameters were chosen for optimisation: CNV calling threshold (default = 1.5, alternatives 1.25 and 1.75 tested) and SVD number (default = 2, alternatives 3 - 5 tested).


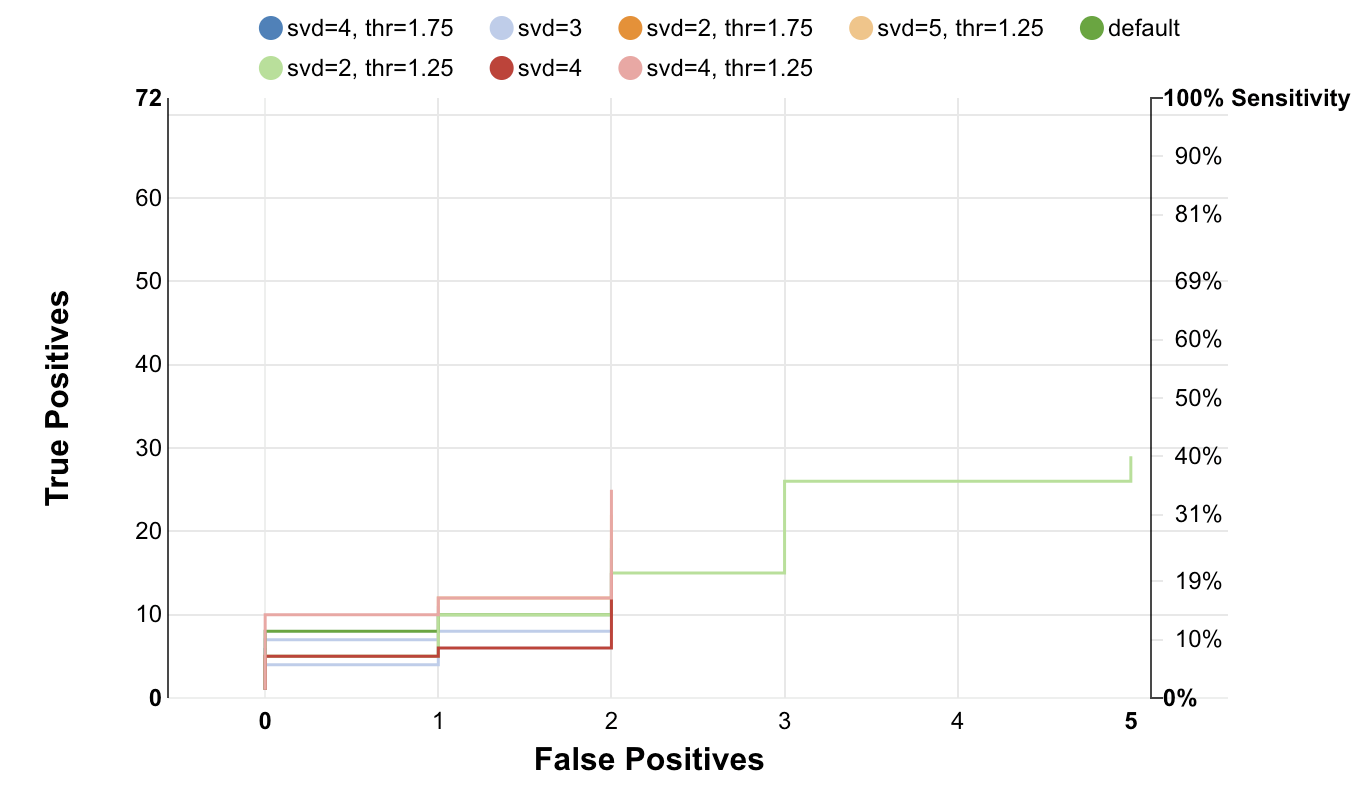


***Figure S5:*** *Optimisation of calling parameters for Conifer. SVD number and calling threshold were adjusted to non-default values (see legend). Changing SVD number to 4 and using threshold 1.25 slightly increased sensitivity, however greater increase was observed with the default SVD number (2) and calling threshold of 1.25.*

### Optimisation of cn.MOPs Calling Parameters

### **Adjustment for Sensitivity**


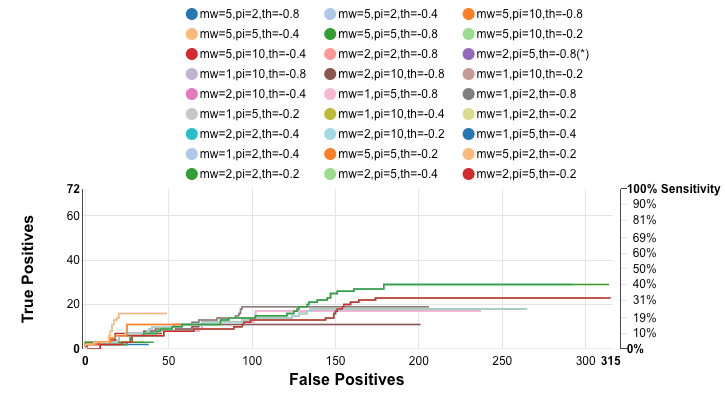


**Figure S6*:*** *Optimisation of calling parameters for cn.MOPs. Three parameters were adjusted through a range of values to find the optimal settings: minimum width (mw) from 1 to 4, prior impact (pi) from 2 to 10, and calling threshold (th) from -0.2 to -0.8. The settings of minimum width=2, prior impact=2 and calling threshold=-0.2 offer nearly double the sensitivity (increase from 24% to 40%), however these settings cause a substantial degradation in precision.*

### Adjustment for Quality Control


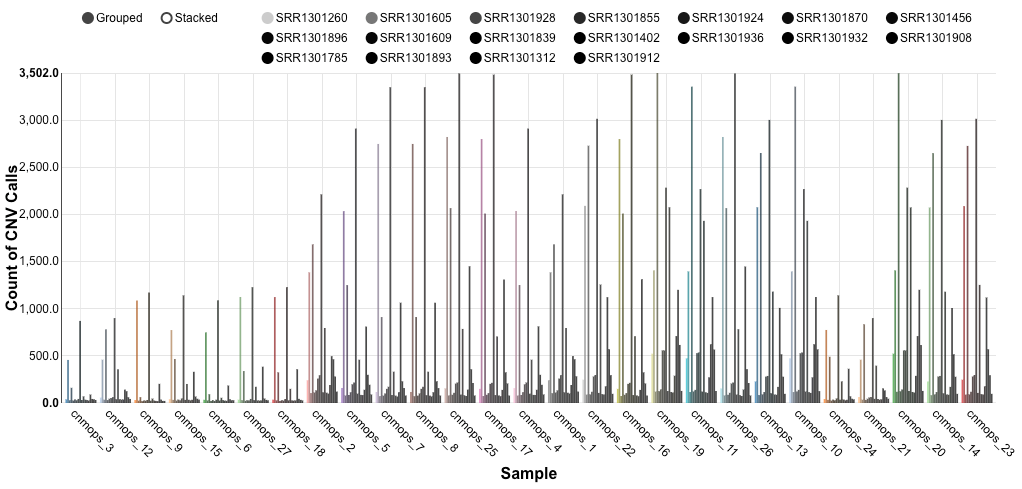


**Figure S7*:*** *Sample count QC plot showing disproportionate numbers of CNV calls concentrated in 3 particular samples across all parameter settings. Each separate configuration of cn.MOPs is represented by a separate group of bars along the x-axis. Within each group, three bars can be seen to significantly exceed the height of other bars, representing poor quality samples within the batch.*

## 3: Ximmer Configurations

The following sections show the configurations used when running Ximmer to analyse CNVs and generate the reports and plots referenced in the paper.

### SureSelect Samples

title="Test Simulation"

bam_files="/home/simon.sadedin/work/ximmer/old_simulations/sjia/comparesim0.1/repx/*.bam"

target_regions="/group/bioi1/simons/sjia/design/EXOME.bed"

// We specify "replace" but run with -nosim flag in this case so that

// the code uses pre-defined bams instead of making new ones

simulation_type="replace"

simulation_enabled=false

concurrency=40

enable_parallel_analyses=true

run_directory_prefix = ""

// Not really used: the true positives are specified individually for each run below

known_cnvs="/home/simon.sadedin/work/ximmer/old_simulations/sjia/comparesim0.1/true_cnvs.bed"

// Number of separate runs to complete

// runs=1

draw_cnvs=false

runs {

"comparesim0_1" {

known_cnvs="/home/simon.sadedin/work/ximmer/old_simulations/sjia/comparesim0.1/true_cnvs.bed"

bam_files="/home/simon.sadedin/work/ximmer/old_simulations/sjia/comparesim0.1/xrep/*.bam"

}

"comparesim0_2" {

known_cnvs="/home/simon.sadedin/work/ximmer/old_simulations/sjia/comparesim0.2/true_cnvs.bed"

bam_files="/home/simon.sadedin/work/ximmer/old_simulations/sjia/comparesim0.2/xrep/*.bam"

}

"comparesim3_1" {

known_cnvs="/home/simon.sadedin/work/ximmer/old_simulations/sjia/comparesim3.1/true_cnvs.bed"

bam_files="/home/simon.sadedin/work/ximmer/old_simulations/sjia/comparesim3.1/xrep/*.bam"

}

"comparesim3_2" {

known_cnvs="/home/simon.sadedin/work/ximmer/old_simulations/sjia/comparesim3.2/true_cnvs.bed"

bam_files="/home/simon.sadedin/work/ximmer/old_simulations/sjia/comparesim3.2/xrep/*.bam"

}

"comparesim5_1" {

known_cnvs="/home/simon.sadedin/work/ximmer/old_simulations/sjia/comparesim5.1/true_cnvs.bed"

bam_files="/home/simon.sadedin/work/ximmer/old_simulations/sjia/comparesim5.1/xrep/*.bam"

}

"comparesim5_2" {

known_cnvs="/home/simon.sadedin/work/ximmer/old_simulations/sjia/comparesim5.2/true_cnvs.bed"

bam_files="/home/simon.sadedin/work/ximmer/old_simulations/sjia/comparesim5.2/xrep/*.bam"

}

}

regions=2..10

deletionsPerSample=0

samples {

males = [

]

females = [

"J00013M",

"J00019M",

"J00058M",

"J00060M",

"J00082M",

"J00113M",

"J00141M",

"J00152M",

"J00170Mum",

"J00338M",

"J00367M",

"J00384M",

"J00403M",

"J00463M"

]

}

dgv {

max_freq = 0.01

min_study_size = 5

}

callers {

xhmm {

exome_wide_cnv_rate=1e-08

mean_number_of_targets_in_cnv=3

}

exomedepth { transition_probability=0.0001 }

cnmops { prior_impact=5; min_width=1; lower_threshold=-0.8 }

conifer { conifer_svd_num=2 }

}

analyses {

base {

xhmm { }

exomedepth {}

cnmops {}

conifer {}

}

nimopt {

xhmm { label="xhmm default" }

exomedepth { label="ed default"}

cnmops { label="cnmops default"}

conifer {label="conifer default"}

xhmm_nimopt {

exome_wide_cnv_rate=1e-04;

xhmm_pve_mean_factor=0.2;

label='xhmm opt'

}

exomedepth_nimopt {

transition_probability=0.0001; expected_cnv_length=50000; label='ed opt'

}

conifer_nimpopt {

conifer_call_threshold=1.25; conifer_svd_num=2; label='conifer opt'

}

cnmops_nimpopt {

prior_impact=2

lower_threshold=-0.4

min_width=1

label='cnmops opt'

}

}

}

### Nextera Samples

title="MGHA Samples Simulation"

bam_files="/home/simon.sadedin/work/ximmer/old_simulations/sjia/sim1.1/repx/*.bam"

target_regions="/home/simon.sadedin/work/ximmer/old_simulations/mgha/design/NEXTERA12.bed"

simulation_type="replace"

simulation_enabled=false // pre-simulated data

concurrency=80

enable_parallel_analyses=true

run_directory_prefix = ""

// Not really used: the true positives are specified individually for each run below

known_cnvs="/home/simon.sadedin/work/ximmer/old_simulations/mgha/sim1.1/true_cnvs.bed"

draw_cnvs = false

runs {

"sim1_1" {

known_cnvs="/home/simon.sadedin/work/ximmer/old_simulations/mgha/sim1.1/true_cnvs.bed"

bam_files="/home/simon.sadedin/work/ximmer/old_simulations/mgha/sim1.1/xrep/*.bam"

}

"sim1_2" {

known_cnvs="/home/simon.sadedin/work/ximmer/old_simulations/mgha/sim1.2/true_cnvs.bed"

bam_files="/home/simon.sadedin/work/ximmer/old_simulations/mgha/sim1.2/xrep/*.bam"

}

"sim3_1" {

known_cnvs="/home/simon.sadedin/work/ximmer/old_simulations/mgha/sim3.1/true_cnvs.bed"

bam_files="/home/simon.sadedin/work/ximmer/old_simulations/mgha/sim3.1/xrep/*.bam"

}

"sim3_2" {

known_cnvs="/home/simon.sadedin/work/ximmer/old_simulations/mgha/sim3.2/true_cnvs.bed"

bam_files="/home/simon.sadedin/work/ximmer/old_simulations/mgha/sim3.2/xrep/*.bam"

}

"sim5_1" {

known_cnvs="/home/simon.sadedin/work/ximmer/old_simulations/mgha/sim5.1/true_cnvs.bed"

bam_files="/home/simon.sadedin/work/ximmer/old_simulations/mgha/sim5.1/xrep/*.bam"

}

"sim5_2" {

known_cnvs="/home/simon.sadedin/work/ximmer/old_simulations/mgha/sim5.2/true_cnvs.bed"

bam_files="/home/simon.sadedin/work/ximmer/old_simulations/mgha/sim5.2/xrep/*.bam"

}

}

regions=2..10

deletionsPerSample=0

samples {

males = [

]

females = [

"S1019656043",

"S1145112076",

"S1193780528",

"S1385464432",

"S1584709162",

"S1871153506",

"S2042983276",

"S2187557630",

"S4143438373",

"S5139042137",

"S5234011865",

"S5703141464",

"S6053331460",

"S6118691544",

"S6213941212",

"S6242296911",

"S6657039395",

"S6820962506",

"S7174069399",

"S7386041098",

"S7418863373",

"S7710680155",

"S8288511570",

"S8950112496"

]

}

dgv {

max_freq = 0.01

min_study_size = 5

}

callers {

xhmm {

exome_wide_cnv_rate=1e-08

mean_number_of_targets_in_cnv=3

}

exomedepth { transition_probability=0.0001 }

cnmops { prior_impact=5; min_width=1; lower_threshold=-0.8 }

conifer { conifer_svd_num=2 }

}

analyses {

base {

xhmm { }

exomedepth {}

cnmops {}

conifer {}

}

nimopt {

xhmm { label="xhmm default" }

exomedepth { label="ed default"}

cnmops { label="cnmops default"}

conifer {label="conifer default"}

xhmm_nimopt {

exome_wide_cnv_rate=1e-04;

xhmm_pve_mean_factor=0.2;

label='xhmm opt'

}

exomedepth_nimopt {

transition_probability=0.0001; expected_cnv_length=50000; label='ed opt'

}

conifer_nimpopt {

conifer_call_threshold=1.25; conifer_svd_num=2; label='conifer opt'

}

cnmops_nimpopt {

prior_impact=2

lower_threshold=-0.4

min_width=1

label='cnmops opt'

}

}

}

### TruSeq Samples

title="CNV Evaluation for Broad Capture"

// bam_files="/home/simon.sadedin/work/ma/vcgs/crams/*.cram"

bam_files="/home/simon.sadedin/work/ximmer/configs/broad/crams/*.cram"

// Note: these target regions are different to the original regions

// that were actually the exome variant calling regions

// Those were nearly 60mb and extend a long way outside the regions targeted

// by the capture kit.

target_regions="/home/simon.sadedin/work/ma/vcgs/target_regions.bed"

// We specify "replace" but run with -nosim flag in this case so that

// the code uses pre-defined bams instead of making new ones

simulation_type="replace"

concurrency=28

enable_parallel_analyses=true

run_directory_prefix = ""

// Not really used: the true positives are specified individually for each run below

// known_cnvs="/home/simon.sadedin/work/ximmer/old_simulations/sjia/comparesim0.1/true_cnvs.bed"

// Number of separate runs to complete

runs=2

regions=2..10

deletionsPerSample=3

ped_file="/home/simon.sadedin/work/ximmer/configs/broad/crams/vcgs_samples.ped"

draw_cnvs=false

dgv {

max_freq = 0.01

min_study_size = 5

}

callers {

xhmm {

exome_wide_cnv_rate=1e-08

mean_number_of_targets_in_cnv=3

}

exomedepth { transition_probability=0.0001 }

cnmops { prior_impact=5; min_width=1; lower_threshold=-0.8 }

conifer { conifer_svd_num=2 }

}

analyses {

base {

xhmm { }

exomedepth {}

cnmops {}

conifer {}

}

nimopt {

xhmm { label="xhmm default" }

exomedepth { label="ed default"}

cnmops { label="cnmops default"}

conifer {label="conifer default"}

xhmm_nimopt {

exome_wide_cnv_rate=1e-04;

xhmm_pve_mean_factor=0.2;

label='xhmm opt'

}

exomedepth_nimopt {

transition_probability=0.0001; expected_cnv_length=50000; label='ed opt'

}

conifer_nimpopt {

conifer_call_threshold=1.25; conifer_svd_num=2; label='conifer opt'

}

cnmops_nimpopt {

prior_impact=2

lower_threshold=-0.4

min_width=1

label='cnmops opt'

}

}

}

### Tuning of Nimblegen Samples

title="Simulation of CNV Detection in Simons Simplex Exomes (X replacement)"

bam_files="/group/bioi1/simons/cpipe6/batches/simplex/analysis/align/*.recal.bam"

target_regions="/group/bioi1/simons/cpipe6/batches/simplex/design/NIMBLEGENV2.bed"

simulation_type="replace"

concurrency=100

// Number of separate runs to complete

runs=2

regions=4..15

deletionsPerSample=2

enable_parallel_analyses=true

draw_cnvs=false

samples {

males = [

"SRR1301236", "SRR1272287", "SRR1272253", "SRR1301293"

]

females = [

"SRR1301839",

"SRR1301908",

"SRR1301932",

"SRR1301896",

"SRR1301260",

"SRR1301870",

"SRR1301936",

"SRR1301912",

"SRR1301312",

"SRR1301605",

"SRR1301928",

"SRR1301609",

"SRR1301924",

"SRR1301855",

"SRR1301785",

"SRR1301893",

"SRR1301402",

"SRR1301456"

]

}

dgv {

max_freq = 0.05

min_study_size = 5

}

callers {

xhmm {

exome_wide_cnv_rate=1e-08

mean_number_of_targets_in_cnv=3

}

exomedepth { transition_probability=0.0001 }

cnmops { prior_impact=5; min_width=1; lower_threshold=-0.8 }

conifer { conifer_svd_num=2 }

codex { codex_k=0 }

}

cnmops_param_values = [

min_width: [1,2,5],

prior_impact: [2,5,10],

lower_threshold: [-0.8,-0.4,-0.2]

]

def cnmops_defaults = [

min_width: 2, // note: actual min width default in settings = 5

prior_impact: 5,

lower_threshold: -0.8

]

def i=0

def cnmops_params = cnmops_param_values*.value.combinations().collect {

[

index: ++i,

min_width: it[0],

prior_impact: it[1],

lower_threshold: it[2]

]

}

analyses {

codex_tun {

codex_1 { codex_k=-2; label='k_adj=-2' }

codex_2 { codex_k=-1; label='k_adj=-1' }

codex_3 { codex_k=0; label='default' }

codex_4 { codex_k=1; label='k_adj=1' }

codex_5 { codex_k=2; label='k_adj=2' }

codex_6 { codex_k=3; label='k_adj=3' }

}

'xhmmtun' {

xhmm_1 { exome_wide_cnv_rate=1e-02; label='cnv rate=10⁻², mf=0.7' }

xhmm_2 { exome_wide_cnv_rate=1e-02; xhmm_pve_mean_factor=0.2; label='cnv rate=10⁻², mf=0.2'}

xhmm_3 { exome_wide_cnv_rate=1e-04; label='cnv rate=10⁻⁴, mf=0.7' }

xhmm_4 { exome_wide_cnv_rate=1e-04; xhmm_pve_mean_factor=0.2; label='cnv rate=10⁻⁴, mf=0.2' }

xhmm_5 { exome_wide_cnv_rate=1e-06; label='cnv rate=10⁻⁶, mf=0.7' }

xhmm_6 { exome_wide_cnv_rate=1e-06; xhmm_pve_mean_factor=0.2; label='cnv rate=10⁻⁶, mf=0.2' }

xhmm_defaults { exome_wide_cnv_rate=1e-08; label='default' }

}

'edtun' {

exomedepth_1 { transition_probability=0.000001; expected_cnv_length=50000; label='txpr=10⁻⁶, len=50kbp' }

exomedepth_2 { transition_probability=0.00001; expected_cnv_length=50000; label='txpr=10⁻⁵, len=50kbp' }

exomedepth_3 { transition_probability=0.00001; expected_cnv_length=10000; label='txpr=10⁻⁵, len=10kbp' }

exomedepth_4 { transition_probability=0.0001; expected_cnv_length=50000; label='default' }

exomedepth_5 { transition_probability=0.001; expected_cnv_length=50000; label='txpr=10⁻³, len=50kbp' }

exomedepth_6 { transition_probability=0.001; expected_cnv_length=10000; label='txpr=10⁻³, len=10kbp' }

exomedepth_7 { transition_probability=0.01; expected_cnv_length=50000; label='txpr=10⁻², len=50kbp' }

exomedepth_8 { transition_probability=0.01; expected_cnv_length=10000; label='txpr=10⁻², len=10kbp' }

}

conifersvd {

conifer_svd1 { conifer_svd_num=1 }

conifer_svd2 { conifer_svd_num=2 }

conifer_svd3 { conifer_svd_num=4 }

conifer_svd4 { conifer_svd_num=6 }

conifer_svd5 { conifer_svd_num=8 }

}

coniferthr {

conifer_defaults { conifer_svd_num=2; conifer_call_threshold=1.5; label='default' }

conifer_thr15svd4 { conifer_svd_num=4; conifer_call_threshold=1.5; label='svd=4' }

conifer_thr15svd3 { conifer_svd_num=3; conifer_call_threshold=1.5; label='svd=3' }

conifer_thr125svd4 { conifer_svd_num=4; conifer_call_threshold=1.25; label='svd=4, thr=1.25' }

conifer_thr125svd2 { conifer_svd_num=2; conifer_call_threshold=1.25; label='svd=2, thr=1.25'}

conifer_thr125svd5 { conifer_svd_num=5; conifer_call_threshold=1.25; label='svd=5, thr=1.25' }

conifer_thr175svd4 { conifer_svd_num=4; conifer_call_threshold=1.75; label='svd=4, thr=1.75' }

conifer_thr175svd2 { conifer_svd_num=2; conifer_call_threshold=1.75; label='svd=2, thr=1.75' }

}

cnmopstun {

for(params in cnmops_params) {

"cnmops_${params.index}" {

min_width=params.min_width

prior_impact=params.prior_impact

lower_threshold=params.lower_threshold

label="mw=$params.min_width,pi=$params.prior_impact,th=$params.lower_threshold"

if((params - [index:params.index]) == cnmops_defaults) {

label+="(*)"

}

}

}

}

cnmopsthr {

cnmops_1 { lower_threshold=-0.8; label='lthr=-0.8 (*)'}

cnmops_2 { lower_threshold=-0.6; label='lthr=-0.6'}

cnmops_3 { lower_threshold=-0.4; label='lthr=-0.4'}

cnmops_4 { lower_threshold=-0.2; label='lthr=-0.2'}

}

cnmopspi {

cnmops_1 { prior_impact=10; label='pi=10'}

cnmops_2 { prior_impact=5; label='pi=5 (*)'}

cnmops_3 { prior_impact=2; label='pi=2'}

cnmops_4 { prior_impact=1; label='pi=1'}

}

cnmopsmw {

cnmops_mw1 { min_width=1; label='mw=1 (*)'}

cnmops_mw2 { min_width=2; label='mw=2' }

cnmops_mw3 { min_width=3; label='mw=3' }

cnmops_mw4 { min_width=4; label='mw=4' }

cnmops_mw5 { min_width=4; label='mw=5' }

}

}

### Nimblegen Comparison on Validated Samples

title="Simons Simplex Tuned vs Untuned"

bam_files="/group/bioi1/simons/cpipe6/batches/simplex/analysis/align/*.recal.bam"

target_regions="/group/bioi1/simons/cpipe6/batches/simplex/design/NIMBLEGENV2.bed"

simulation_type="none"

concurrency=180

// Specify this

// ped_file="eval/data/haloplex/haloplex.ped"

known_cnvs="/home/simon.sadedin/work/ximmer/eval/k_e_cnvs.subset.tsv"

// Number of separate runs to complete

runs=1

regions=2..10

deletionsPerSample=0

draw_cnvs=false

samples {

males = [

"SRR1301788", "SRR1301320", "SRR1301717", "SRR1301697", "SRR1301356", "SRR1301885", "SRR1301916", "SRR1301561", "SRR1301352", "SRR1301585", "SRR1301256", "SRR1301679", "SRR1301364", "SRR1301348", "SRR1301613"

]

females = [

"SRR1301839",

"SRR1301908",

"SRR1301932",

"SRR1301896",

"SRR1301260",

"SRR1301870",

"SRR1301936",

"SRR1301912",

"SRR1301312",

"SRR1301605",

"SRR1301928",

"SRR1301609",

"SRR1301924",

"SRR1301554",

"SRR1301855",

"SRR1301785",

"SRR1301893",

"SRR1301402",

"SRR1301456"

]

}

dgv {

max_freq = 1.00

min_study_size = 5

}

callers {

xhmm {

exome_wide_cnv_rate=1e-08

mean_number_of_targets_in_cnv=3

}

exomedepth { transition_probability=0.0001 }

cnmops { prior_impact=10; min_width=5; lower_threshold=-0.8; upper_threshold=0.55 }

conifer { conifer_svd_num=1 }

}

bad_cnmops_samples=[

'SRR1301609',

'SRR1301855',

'SRR1301605'

]

analyses {

base {

xhmm { }

exomedepth {}

cnmops {}

conifer {}

}

opt2 {

/**

* Defaults

*/

xhmm { label="xhmm default" }

exomedepth { label="ed default"}

conifer {label="conifer default"}

cnmops { label="cnmops default"}

/**

* Optimised

*/

xhmm_opt {

exome_wide_cnv_rate=1e-04;

xhmm_pve_mean_factor=0.2;

label='xhmm opt'

}

exomedepth_opt {

transition_probability=0.0001; expected_cnv_length=50000; label='ed opt'

}

conifer_opt {

conifer_call_threshold=1.25; conifer_svd_num=2; label='conifer opt'

}

cnmops_excl {

label="exclude 3"

exclude_samples=bad_cnmops_samples

}

}
