# Supplementary material for: Ximmer: a system for improving accuracy and consistency of CNV calling from exome data
Source: Gigascience. 2018 Sep 6;7(10):giy112. doi: 10.1093/gigascience/giy112 (PMC6177737; doi:10.1093/gigascience/giy112)
Supplement: Response_to_Reviewer_Comments_Original_Submission.pdf [file giy112_response_to_reviewer_comments_original_submission.pdf]

Author's Response To Reviewer Comments

Close

Reviewer #1:

> To detect copy number variation (CNV) by whole-exome sequencing (WES) is non-trivial, due to the biases and artifacts that introduced during library prep and sequencing. Furthermore, previously developed methods reported discordant benchmark results and show significant variability in performance for real dataset analysis. Sadedin et al. proposed Ximmer, a bioinformatic pipeline for detecting CNV by WES, which includes a simulation method, an analysis pipeline, and a graphical report. My comments are below:

> 1) The authors made the claim that depleting reads is significantly simpler than synthesising and adding new reads and thus focused on spiking in deletions. However, deletions are also easier to detect than duplications besides the tuning for deletions might not work the same way for duplications. I am not sure why the linear assumption between copy number and read depth in autosomes won't hold for duplications and thus why the authors only spike in deletions as gold standards. This linear relationship is a very strong assumption and should be checked empirically using experimentally validated CNVs.

We acknowledge that extension to duplications would enhance the utility of Ximmer. Our reason for omitting this functionality is not due to the linearity assumption, but rather the complexity of simulating new reads and adding them to an existing alignment in a realistic manner. We have rephrased the section describing this, and also added a sentence to the conclusion to mention that extension to duplications would be valuable future work.

> 2) Deletions are randomly spiked in along the genome. What if they overlap with existing true CNVs? This will be extremely prominent in cancer samples where large chromosomal changes are observed.

We have added clarifying text to the introduction and abstract to emphasise that currently Ximmer is intended for use with germline and not cancer samples. To reduce the possibility of overlap with real CNVs, Ximmer avoids simulating in regions that overlap with events in the Database of Genomic Variants (DGV), a well known database of population CNVs. Addition of other public CNV databases is planned for future versions of Ximmer.

> 3) The lengths and population frequencies for the added deletion will affect performance. This needs to be further evaluated and clarified.

As noted above, we ensure that only very rare CNVs will overlap simulated deletions. Ximmer includes features to help understand performance of differing CNV sizes. We have added the following clarifying text to the section on Accuracy Assessment to better highlight these features:

“However, it is frequently of interest to know how sensitivity varies for CNVs of different sizes. The Ximmer accuracy plot can be interactively adjusted, to show performance of a subset of CNVs within size ranges specified in base-pairs or number of target regions.

Further, the accuracy plot can also show the performance of combinations of results such as the intersection or union of results from different CNV callers."

> 4) The authors declared that there are five commonly used tools integrated in Ximmer but I don't see results from CODEX.

CODEX was published more recently than the other CNV callers included in the manuscript, and therefore we had not, at the time of the original drafting, created results on all of our data sets using it. We have now run CODEX and have added results for it to Figure 3 and Figure 4. As it includes an internal algorithm for optimising its parameters, CODEX does not expose external parameters for tuning in the way that other methods do. Therefore we have omitted CODEX from the section of the manuscript relevant to tuning CNV calling parameters.

> 5) The authors made the claim that different methods tend to have discordant performance assessment results and in the dataset analysis return distinct CNV calls. Ximmer is proposed to improve upon this. However, Ximmer is applied to tune each method individually (Figure 7). How results are concatenated and filtered across callers (not an easy task) remains untapped and unsolved.

We have added text to the section titled "CNV Discovery" to better highlight Ximmer's features for CNV filtering and interpretation. These include a report showing merged CNV results where multiple overlapping CNV calls are combined together into a single result. The table can be filtered based on individual caller quality scores among other parameters, helping to address this issue.

> 6) The authors need to more clearly define and specify the parameters that need to be optimized across methods, as these will still need to manually tuned by the users.

The parameters that we selected for optimization are listed in Table S2 (Supplementary Material). We outline which parameters were effective in our tested data sets in the section titled "CNV Calling performance can be improved with parameter optimisation". However we believe that determination of the most effective parameters is likely to be data set specific, and therefore recommend use of Ximmer to discover these.

> 7) What is the predicted sensitivity in Table 2?

The predicted sensitivity is the sensitivity estimated by Ximmer from simulation results. We have expanded the caption of this table to make this clearer.

Reviewer #2:

>The paper presents a new approach called Ximmer for detecting of copy number variations from exome sequencing data. The main contribution of the paper is using different CNV detection methods to optimize copy number variation detection performance and improving

the accuracy of WES-based CNV detection methods. The results show that the method is effective after tuning parameters of tools. The paper is well written and the approach are shown with nice plots. There is a typo 'depleted' in manuscript.
> 1- Why did the authors choose these 4 tools among all of WES based CNV detection tools?

We have added the following text to the section titled “CNV Analysis Pipeline” to address this question:

“These tools were selected by surveying the literature to ascertain popular methods that are applicable to germline CNV detection. The set was then narrowed to those that were empirically found to be straightforward to install and run reliably within Ximmer’s automated framework. We expect to add further tools over time as new methods become available.”

> 2- The authors used threshold to call CNVs. What is the average differences between detected CNVs and benchmark CNVs values before and after tuning?

Ximmer does not itself use thresholds in calling CNVs, but rather relies on the algorithms of the individual CNV detection methods, some of which apply thresholds while others apply statistical confidence measures. We calculated that there was a slight decrease in difference between benchmark and false CNVs for XHMM (2%) after tuning and a slight increase in confidence measure difference (2%) for Conifer. ExomeDepth and cn.MOPS were unchanged. We point out however that we do not optimise for this metric in our manuscript, only for improvement in overall sensitivity and precision.

Reviewer #3:

>The manuscript "Ximmer: A System for Improving Accuracy and Consistency of CNV Calling from Exome Data" presents a new method to detect copy number variations (CNV) using whole exome sequencing (WES) data. The manuscript is clear to read and the work is sound and coherent. As WES is widely used and CNV detection methods from WES data are lacking, such studies are important and timely. The effort to make usage easier to use and visualize the results is appreciated. I suggest the following to further improve the work:
> 1. Although the authors briefly describe other equivalent methods, it is important to compare these methods (other than only statistically) by their properties, algorithm, usage, resources etc., possibly in a table or by discussion.

We have added a table to the background section of the manuscript that gives an overview of the underlying properties of the methods.

> 2. It would be useful to know the performance of Ximmer for detecting heterozygous, hemizygous and homozygous CNVs, which can be a bottleneck in detecting CNVs from WES data.

As we have already made clear in the manuscript, Ximmer itself does not detect CNVs, rather it depends on the characteristics of the CNV detection methods that are included. We have extensively characterised the detection of heterozygous deletions in the manuscript. We have observed however that hemizygous and homozygous CNVs are significantly easier to detect than heterozygous events, and therefore are not typically limiting factors in

performance. We have expanded the discussion in the conclusion of the manuscript to address these points.

> 3. Discussing (or better, estimating) Ximmer in whole genome sequencing data (WGS) would be important for users of WGS data.

While there are many methods to call CNVs on WGS data, these tools operate by very different principles which in turn require very different underlying methods for simulation. Furthermore, the additional signals available to WGS algorithms make the problem much more tractable. For these reasons we believe that a simulation and evaluation tool dedicated to CNV detection on exomes is warranted.

Reviewer #4:

> In this manuscript, authors proposed a tool (Ximmer) that can evaluate, tune and run exome CNV tools. They evaluated four CNV tools using data sets from four different platforms. Ximmer can optimize automatically running parameters to achieve the best performance. However, I am wondering whether Ximmer is actually useful when CNV analysis is performed using inputs of own datasets. Because own datasets have different setting such as read depth, sample size and sample types. In addition, the manuscript does not provide clear threshold about which samples pass or fail quality control (QC).

Thank you for your comments. We agree that the difficulty of addressing variable sequencing parameters is one of the main challenges in CNV detection from exome data. One of the key features of Ximmer and our motivation for developing it is to allow users to understand performance and tune the methods with their own data.

> In Table 2, sensitivity of XHMM and Conifer was decreased after the parameter optimization. If their performances are lower than those of default setting, why tuning step is needed?

The caption on Table 2 was unclear and may have contributed to a misinterpretation of the figures: the decrease shown in the table is actually relative to Ximmer’s prediction of sensitivity. That is, Ximmer slightly overestimated the sensitivity prior to tuning. After tuning, sensitivity was increased. We have adjusted the caption to make this clearer.

> There are many weak points regarding the quality of the description and plots:
> * I am not sure which three samples have poor quality in Figure S6. There is no Figure S7.

The headings and plots have been adjusted to make Figure S6 and Figure S7 clearer. Text has been added to the caption of Figure S7 to better identify the three poor quality samples.

> * In Figure 2A, it needs to describe what 'grouped and stacked' means.

The caption has been improved to explain these options.

> * In Figure 2B, each line of frequency CNV calls is not distinguishable.

We acknowledge that the clarity of this figure is not optimal, however the figure is intended to be illustrative of the general appearance of the interface rather than to convey the precise data. Text has been added to the caption inviting readers to visit http://example.ximmer.org where this plot can be viewed in the live interface at full resolution.

> * The explanation of quality score calibration plot (in Figure 2C) should be clarified. It is difficult to understand why quality scores have negative values? QC seems to be confidence measure from each caller. If a unified QC measure is shown, no matter what kind of tool is used, it would be better to interpret the relationship between precision and QC more clearly.

The quality scores are indeed derived from each caller, and in some cases these allow for negative values. Although we could rescale them to a positive range, we feel it is important to keep the displayed values consistent with those documented for use with the tool.

> * The manuscript describes that 'the cn.MOPs minimum CNV width was lowered to 1'. In manual of cn.MOPs, minimum CNV width is 3.

This sentence has been reworded to make it clearer that we modified the minimum width setting away from the value specified in the manual.

> * In Results, "Increased XHMM sensitivity (Figure 5A)…" should be modified. This corresponds to Figure 5B.

The figure reference has been corrected.

Close
