# Supplementary material for: Ximmer: a system for improving accuracy and consistency of CNV calling from exome data
Source: Gigascience. 2018 Sep 6;7(10):giy112. doi: 10.1093/gigascience/giy112 (PMC6177737; doi:10.1093/gigascience/giy112)
Supplement: Reviewer_1_Report_(Original_Submission) -- Yuchao Jiang — 6/6/2018 Reviewed [file giy112_reviewer_1_report_(original_submission).pdf]

Reviewer Report

Title: Ximmer: A System for Improving Accuracy and Consistency of CNV Calling from Exome Data

Version: Original Submission **Date:** 6/6/2018

Reviewer name: Yuchao Jiang

Reviewer Comments to Author:

To detect copy number variation (CNV) by whole-exome sequencing (WES) is non-trivial, due to the biases and artifacts that introduced during library prep and sequencing. Furthermore, previously developed methods reported discordant benchmark results and show significant variability in performance for real dataset analysis. Sadedin et al. proposed Ximmer, a bioinformatic pipeline for detecting CNV by WES, which includes a simulation method, an analysis pipeline, and a graphical report. My comments are below:

1) The authors made the claim that depleting reads is significantly simpler than synthesising and adding new reads and thus focused on spiking in deletions. However, deletions are also easier to detect than duplications besides the tuning for deletions might not work the same way for duplications. I am not sure why the linear assumption between copy number and read depth in autosomes won't hold for duplications and thus why the authors only spike in deletions as gold standards. This linear relationship is a very strong assumption and should be checked empirically using experimentally validated CNVs.

2) Deletions are randomly spiked in along the genome. What if they overlap with existing true CNVs? This will be extremely prominent in cancer samples where large chromosomal changes are observed.

3) The lengths and population frequencies for the added deletion will affect performance. This needs to be further evaluated and clarified.

4) The authors declared that there are five commonly used tools integrated in Ximmer but I don't see results from CODEX.

5) The authors made the claim that different methods tend to have discordant performance assessment results and in the dataset analysis return distinct CNV calls. Ximmer is proposed to improve upon this. However, Ximmer is applied to tune each method individually (Figure 7). How results are concatenated and filtered across callers (not an easy task) remains untapped and unsolved.

6) The authors need to more clearly define and specify the parameters that need to be optimized across methods, as these will still need to manually tuned by the users.

7) What is the predicted sensitivity in Table 2?

Level of Interest

Please indicate how interesting you found the manuscript: Choose an item.

Quality of Written English

Please indicate the quality of language in the manuscript: Choose an item.

Declaration of Competing Interests

Please complete a declaration of competing interests, considering the following questions:

- Have you in the past five years received reimbursements, fees, funding, or salary from an organisation that may in any way gain or lose financially from the publication of this manuscript, either now or in the future?
- Do you hold any stocks or shares in an organisation that may in any way gain or lose financially from the publication of this manuscript, either now or in the future?
- Do you hold or are you currently applying for any patents relating to the content of the manuscript?
- Have you received reimbursements, fees, funding, or salary from an organization that holds or has applied for patents relating to the content of the manuscript?
- Do you have any other financial competing interests?
- Do you have any non-financial competing interests in relation to this paper?

If you can answer no to all of the above, write 'I declare that I have no competing interests' below. If your reply is yes to any, please give details below.

I declare that I have no competing interests

I agree to the open peer review policy of the journal. I understand that my name will be included on my report to the authors and, if the manuscript is accepted for publication, my named report including any attachments I upload will be posted on the website along with the authors' responses. I agree for my report to be made available under an Open Access Creative Commons CC-BY license (http://creativecommons.org/licenses/by/4.0/). I understand that any comments which I do not wish to be included in my named report can be included as confidential comments to the editors, which will not be published.

I agree to the open peer review policy of the journal

To further support our reviewers, we have joined with Publons, where you can gain additional credit to further highlight your hard work (see: https://publons.com/journal/530/gigascience). On publication of this paper, your review will be automatically added to Publons, you can then choose whether or not to claim your Publons credit. I understand this statement. Yes
