# Supplementary material for: Ximmer: a system for improving accuracy and consistency of CNV calling from exome data
Source: Gigascience. 2018 Sep 6;7(10):giy112. doi: 10.1093/gigascience/giy112 (PMC6177737; doi:10.1093/gigascience/giy112)
Supplement: Reviewer_2_Report_(Original_Submission) -- Fatemeh Zare — 7/6/2018 Reviewed [file giy112_reviewer_2_report_(original_submission).pdf]

Reviewer Report

Title: **Ximmer: A System for Improving Accuracy and Consistency of CNV Calling from Exome Data**

Version: **Original Submission** **Date:** 7/6/2018

Reviewer name: Fatemeh Zare

Reviewer Comments to Author:

The paper presents a new approach called Ximmer for detecting of copy number variations from exome sequencing data. The main contribution of the paper is using different CNV detection methods to optimize copy number variation detection performance and improving the accuracy of WES-based CNV detection methods. The results show that the method is effective after tuning parameters of tools. The paper is well written and the approach are shown with nice plots. There is a typo 'depleted' in manuscript. The comments: 1- Why did the authors choose these 4 tools among all of WES based CNV detection tools? 2- The authors used threshold to call CNVs. What is the average differences between detected CNVs and benchmark CNVs values before and after tuning?

Level of Interest

Please indicate how interesting you found the manuscript: Choose an item.

Quality of Written English

Please indicate the quality of language in the manuscript: Choose an item.

Declaration of Competing Interests

Please complete a declaration of competing interests, considering the following questions:

- Have you in the past five years received reimbursements, fees, funding, or salary from an organisation that may in any way gain or lose financially from the publication of this manuscript, either now or in the future?
- Do you hold any stocks or shares in an organisation that may in any way gain or lose financially from the publication of this manuscript, either now or in the future?
- Do you hold or are you currently applying for any patents relating to the content of the manuscript?
- Have you received reimbursements, fees, funding, or salary from an organization that holds or has applied for patents relating to the content of the manuscript?
- Do you have any other financial competing interests?
- Do you have any non-financial competing interests in relation to this paper?

If you can answer no to all of the above, write 'I declare that I have no competing interests' below. If your reply is yes to any, please give details below.

I declare that I have no competing interests

I agree to the open peer review policy of the journal. I understand that my name will be included on my report to the authors and, if the manuscript is accepted for publication, my named report including any attachments I upload will be posted on the website along with the authors' responses. I agree for my report to be made available under an Open Access Creative Commons CC-BY license (http://creativecommons.org/licenses/by/4.0/). I understand that any comments which I do not wish to be included in my named report can be included as confidential comments to the editors, which will not be published.

I agree to the open peer review policy of the journal

To further support our reviewers, we have joined with Publons, where you can gain additional credit to further highlight your hard work (see: https://publons.com/journal/530/gigascience). On publication of this paper, your review will be automatically added to Publons, you can then choose whether or not to claim your Publons credit. I understand this statement. Yes
