# Supplementary material for: Ximmer: a system for improving accuracy and consistency of CNV calling from exome data
Source: Gigascience. 2018 Sep 6;7(10):giy112. doi: 10.1093/gigascience/giy112 (PMC6177737; doi:10.1093/gigascience/giy112)
Supplement: Reviewer_4_Report_(Original_Submission) -- Je-Gun Joung — 6/24/2018 Reviewed [file giy112_reviewer_4_report_(original_submission).pdf]

Reviewer Report

Title: Ximmer: A System for Improving Accuracy and Consistency of CNV Calling from Exome Data

Version: Original Submission **Date:** 6/24/2018

Reviewer name: Je-Gun Joung

Reviewer Comments to Author:

In this manuscript, authors proposed a tool (Ximmer) that can evaluate, tune and run exome CNV tools. They evaluated four CNV tools using data sets from four different platforms. Ximmer can optimize automatically running parameters to achieve the best performance. However, I am wondering whether Ximmer is actually useful when CNV analysis is performed using inputs of own datasets. Because own datasets have different setting such as read depth, sample size and sample types. In addition, the manuscript does not provide clear threshold about which samples pass or fail quality control (QC).In Table 2, sensitivity of XHMM and Conifer was decreased after the parameter optimization. If their performances are lower than those of default setting, why tuning step is needed?There are many weak points regarding the quality of the description and plots: * I am not sure which three samples have poor quality in Figure S6. There is no Figure S7.* In Figure 2A, it needs to describe what 'grouped and stacked' means.* In Figure 2B, each line of frequency CNV calls is not distinguishable. * The explanation of quality score calibration plot (in Figure 2C) should be clarified. It is difficult to understand why quality scores have negative values? QC seems to be confidence measure from each caller. If a unified QC measure is shown, no matter what kind of tool is used, it would be better to interpret the relationship between precision and QC more clearly. * The manuscript describes that 'the cn.MOPs minimum CNV width was lowered to 1'. In manual of cn.MOPs, minimum CNV width is 3. * In Results, "Increased XHMM sensitivity (Figure 5A)…" should be modified. This corresponds to Figure 5B.

Level of Interest

Please indicate how interesting you found the manuscript: Choose an item.

Quality of Written English

Please indicate the quality of language in the manuscript: Choose an item.

Declaration of Competing Interests

Please complete a declaration of competing interests, considering the following questions:

- Have you in the past five years received reimbursements, fees, funding, or salary from an organisation that may in any way gain or lose financially from the publication of this manuscript, either now or in the future?
- Do you hold any stocks or shares in an organisation that may in any way gain or lose financially from the publication of this manuscript, either now or in the future?
- Do you hold or are you currently applying for any patents relating to the content of the manuscript?

- Have you received reimbursements, fees, funding, or salary from an organization that holds or has applied for patents relating to the content of the manuscript?
- Do you have any other financial competing interests?
- Do you have any non-financial competing interests in relation to this paper?

If you can answer no to all of the above, write 'I declare that I have no competing interests' below. If your reply is yes to any, please give details below.

I declare that I have no competing interests

I agree to the open peer review policy of the journal. I understand that my name will be included on my report to the authors and, if the manuscript is accepted for publication, my named report including any attachments I upload will be posted on the website along with the authors' responses. I agree for my report to be made available under an Open Access Creative Commons CC-BY license (http://creativecommons.org/licenses/by/4.0/). I understand that any comments which I do not wish to be included in my named report can be included as confidential comments to the editors, which will not be published.

I agree to the open peer review policy of the journal

To further support our reviewers, we have joined with Publons, where you can gain additional credit to further highlight your hard work (see: https://publons.com/journal/530/gigascience). On publication of this paper, your review will be automatically added to Publons, you can then choose whether or not to claim your Publons credit. I understand this statement. Yes
